# AliFilter: a machine learning approach to alignment filtering

**DOI:** 10.1093/molbev/msag097

**Published:** 2026-04-10

**Authors:** Giorgio Bianchini, Rui Zhu, Francesco Cicconardi, Edmund R R Moody

**Affiliations:** School of Geographical Sciences, University of Bristol, Bristol, UK; Department of Life Sciences, University of Bath, Bath, UK; School of Geographical Sciences, University of Bristol, Bristol, UK; School of Biological Sciences, University of Bristol, Bristol, UK; Departament de Genètica, Microbiologia I Estadística, Universitat de Barcelona, Barcelona, Spain

**Keywords:** multiple sequence alignment, filtering, trimming, machine learning, phylogenetics, comparative genomics

## Abstract

Multiple sequence alignments are a crucial step in many bioinformatic and computational biology analyses, from protein structure and function prediction to the inference of phylogenetic trees. However, highly divergent sequence alignments often contain a significant amount of noise. Reducing noise is normally achieved by filtering the alignment to remove columns that are poorly aligned or offer minimal useful information—either automatically using various software tools or through manual inspection. Manual approaches are labor-intensive and less reproducible but can utilize the researcher's specialist knowledge, rather than relying on filtering criteria that might not be adequate for each alignment. AliFilter bridges these two approaches to alignment curation, using machine learning to automate manual alignment filtering. AliFilter uses a supervised learning approach to create a model from a small number of manually annotated alignments, then applies this model to reproduce the manual annotation on different datasets. Users can employ the program with a default model or create customized models for individual datasets or filtering criteria. AliFilter accurately reproduces the results of manual annotation (98% accuracy) while being resilient to mistakes in the training data. In a typical phylogenomic workflow, AliFilter reduced the runtime by 35% and produced results that were almost identical to the full alignment, unlike other filtering tools we tested. AliFilter is free and open-source software; it is written in C# and distributed under a GPLv3 license from https://github.com/arklumpus/AliFilter, where both the source code and standalone executables for Windows, macOS, and Linux are available.

## Introduction

Multiple sequence alignments (MSAs) are a key tool in computational biology and bioinformatics, enabling protein structure inference with deep-learning approaches (Baek et al. 2021; Jumper et al. 2021), phylogenetic inference (Sankoff et al. 1973; Felsenstein 1981, 2004), functional annotation of genes and proteins (George et al. 1996; Haft et al. 2003; Huntemann et al. 2015; Tatusova et al. 2016; Palù et al. 2022), as well as the identification of specific protein domains or motifs (Sonnhammer et al. 1997; Marchler-Bauer et al. 2002). Historically, sequences of nucleotides or amino acids were manually aligned based on similar biochemical properties (Margoliash 1963; Pauling et al. 1963), but over the last few decades, many improvements have been made in this field—thanks to the development of dynamic programming algorithms (Needleman and Wunsch 1970; Sankoff 1972; Lipman and Pearson 1985; Redelings and Suchard 2005), the increased availability of genomic and proteomic data (Burley et al. 2019; Sayers et al. 2021; Mukherjee et al. 2023), improved computational power and optimization (Thompson et al. 1994; Notredame et al. 2000; Katoh et al. 2002; Notredame 2002; Edgar 2004), and the use of phylogenetic or protein structure information (O’Sullivan et al. 2004; Suchard and Redelings 2006; Katoh and Standley 2013; Nawrocki and Eddy 2013; Sievers and Higgins 2014). Thanks to these advancements, modern approaches can infer MSAs for thousands of sequences in a matter of seconds with a standard laptop.

In the current ’Omics era, an ever-increasing number of available genomes and proteomes (https://www.ncbi.nlm.nih.gov/genbank/statistics/) require applications of MSAs. These can be computationally expensive and would benefit from greater efficiency; in addition to reducing computation time, this would reduce carbon emissions and allow analyses to be performed by researchers with limited computational resources. Like many other data sources, MSAs are often a mixture of useful information (“signal”), and various kinds of artefacts (“noise”), introduced by the alignment algorithm or even by the underlying evolutionary processes (e.g. as a result of asymmetric tree shapes, high or differential evolutionary rates, or insertions and deletions that affect local sequence similarity) (Lake 1991; Thompson et al. 1999; Hall 2005; Ogden and Rosenberg 2006; Lunter et al. 2008; Wong et al. 2008). One approach to optimizing MSA-dependent analyses is to maximize the signal/noise ratio by removing parts of the alignment that do not provide useful information. This process is generally referred to as alignment “trimming,” “filtering,” or “cleaning” and can be performed either manually or through various tools and approaches (Castresana 2000; Talavera and Castresana 2007; Dress et al. 2008; Capella-Gutiérrez et al. 2009; Criscuolo and Gribaldo 2010; Steenwyk et al. 2020).

**Figure 1:**
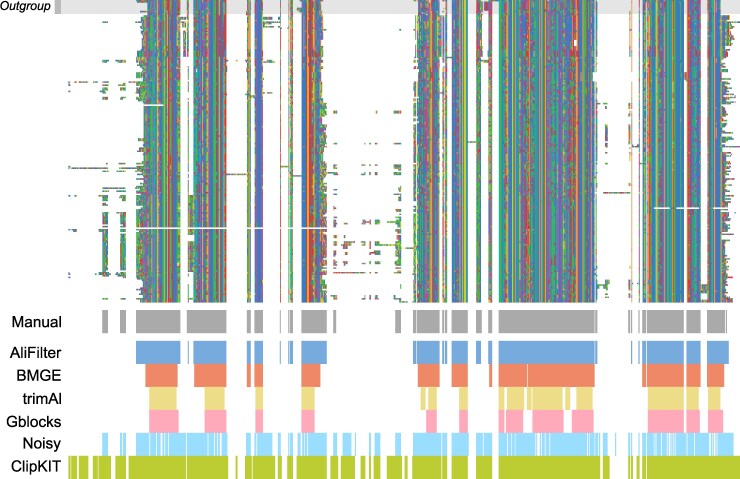
Alignment filtering. The top part of the figure shows a protein sequence alignment for orthologs of the *queA* gene within Cyanobacteria and other terrabacteria (Outgroup). The bottom section shows the results of manual filtering and filtering by various tools; for each approach, unmarked columns were discarded, and colored columns were preserved.

Conceptually, MSAs are matrices of characters, where each row represents an individual sequence, and each column asserts homology between residues in different sequences. A MSA can be filtered by removing entire rows (e.g. partial sequences or non-homologs), entire columns (e.g. hyper-variable loops or sequence-specific insertions), or smaller blocks (e.g. misaligned residues in a region with a large proportion of gaps). Here, we focus on whole-column filtering (Fig. 1); many tools have been developed for this task (Tan et al. 2015), which use different criteria to determine parts of the alignment to preserve or discard. Another option is manual filtering, in which the columns to remove are manually selected by a human expert. While manual filtering is highly time-consuming and less reproducible than automated approaches, a potential advantage is that it leverages the individual researcher's knowledge of the data being analyzed, rather than relying on abstract criteria, which, a priori, may or may not be applicable to a certain dataset. For example, in the alignment shown in Fig. 1, existing automated filtering tools (Castresana 2000; Dress et al. 2008; Capella-Gutiérrez et al. 2009; Criscuolo and Gribaldo 2010; Steenwyk et al. 2020) tend to preserve either much more (ClipKIT and Noisy) or much less (BMGE, trimAl, and Gblocks) information than the manual filtering approach.

The idea of using machine learning to automate manual alignment filtering was first introduced in a previous study (Blouin et al. 2009); this involves gathering a collection of manually filtered alignments and using them to develop a model that will replicate the filtering patterns observed therein. However, this software (MANUEL) and the associated webserver were discontinued and are no longer available. Here, we present AliFilter, a new tool that uses a logistic model to reproduce manual alignment filtering. AliFilter can quickly process large sequence alignments and identify columns that should be preserved or discarded based on implicit criteria “learned” from a training dataset. This makes it possible to create filtering models specifically for different types of data or analysis from a relatively small number of manually filtered alignments.

### AliFilter model

#### Alignment features

AliFilter determines whether alignment columns should be preserved or deleted based on six numerical features (Table 1), which were selected to reflect alignment column characteristics that are used in manual annotation (either consciously or unconsciously). These features are computed for every column in the alignment and used as the input data for the machine learning model. During training, model parameters are estimated by combining the feature values with the “true” preservation status, while during alignment filtering, the output of the model is used to determine which columns to discard. Since the features have the same definition for both nucleotide and amino acid alignments, multiple kinds of data can be used to train a single model; furthermore, models trained on nucleotide sequence data can also be applied to amino acid data, and vice versa.

**Table 1:** **Alignment features computed by AliFilter.** The features are defined for both nucleotide and amino acid alignments; the only difference is the maximum entropy value, i.e. ln(4) for nucleotide data and ln(20) for amino acid data. When computing gap proportions ±1 and ±2 for columns close to the start or end of the alignment, non-existing columns are excluded from the average. For example, the gap proportion (±2) for the first column is the average gap proportion between the first, second, and third columns, while the gap proportion (±1) for the last column is the average gap proportion between the last column and the penultimate column.

Feature	Description
**Gap proportion**	Proportion of sequences that have a gap in the column.
**Percent identity**	Frequency of the most common residue in the alignment column (excluding gaps)
**Distance from extremity**	Number of residues between the column and the closest extremity (start or end) of the alignment.
**Entropy**	Shannon entropy (natural base) for the residue frequencies in the column, excluding gaps.
**Gap proportion (±1)**	Average gap proportion between the column, the preceding column, and the subsequent column.
**Gap proportion (±2)**	Average gap proportion between the column, the two columns preceding it, and the two subsequent columns.

#### Logistic model

The machine learning model implemented in AliFilter is a logistic model (Hosmer and Lemeshow 2000) with seven parameters. Given a set of features, the program computes a preservation score p∈(0,1) for each alignment column:


$$p=\mathrm{logi}t^{-1}\left(\overset{6}{\underset{i=0}{\sum }}\;m_{i}\cdot f_{i}\right)$$

where $m_{0},\;m_{1}\ldots m_{6}$ are the model parameters, $f_{1},\;f_{2}\ldots f_{6}$ are the six features for the alignment column, $f_{0}=1$, and $logit^{-1}(x)=\frac{1}{1+e^{-x}}$. Given a preservation threshold t∈[0,1], columns where p>t are preserved, while columns where p≤t are removed from the alignment. The values of $m_{0},\;m_{1}\ldots m_{6}$ are estimated during the model training step, while the value of *t* is estimated during model validation (for unvalidated models, a default value of 0.5 is used).

The value of *p* provides a measure of how confident the model is in determining whether a single column should be preserved or deleted. It is thus possible to compute an overall confidence score C∈[0,1) for a set of multiple columns (e.g. a whole alignment), defined as:


$$C=1-\frac{4}{n}\overset{n}{\underset{k=1}{\sum }}\;p_{k}\cdot (1-p_{k})$$

where *n* is the total number of columns being considered and $p_{k}$ is the preservation score for column *k*. Each individual column's contribution to this score is symmetrical (i.e. a column with score $p_{k}$ has the same effect as a column with score $1-p_{k}$), and the constant value 4 serves to normalize *C* to the range [0,1); C≈1 when all the columns have very confident assignments (i.e. $p_{k}\approx 1$ or $p_{k}\approx 0$), while C=0 when all the column assignments have maximum uncertainty ($p_{k}=0.5$).

#### Model training, validation, and testing

Creating an AliFilter model follows the standard machine learning process of model training, validation, and testing (Bishop 2006). For each step, AliFilter requires a set of reference alignments and a “mask” for each alignment, which specifies which columns should be preserved and which ones should be deleted. During the model training step, an iteratively reweighted least squares approach (Björck 1996) is used to estimate the model parameters for the logistic model ($m_{0},\;m_{1},\;\ldots m_{6}$), optimizing the log loss function (Murphy 2012).

During the model validation step, AliFilter applies the trained model to the validation dataset and finds the threshold value *t* that results in the best performance according to one of three metrics. The default performance metric is the Matthews correlation coefficient (*MCC*; (Matthews 1975)), which measures the correlation between model assignments and the assignments in the input data. Alternatively, command-line options can be used to switch to the Accuracy metric (*A*) or to the $F_{\beta }$ score for a specific value of *β* (Chinchor 1992). For a fairly balanced validation dataset, such as the one that was used for our analyses, all three metrics should produce very similar results; we refer the reader to (Chicco and Jurman 2020) for a discussion on the merits of each metric.

Bootstrap replicates are also analyzed during the model validation step. Unlike the traditional bootstrap process in phylogenetic analyses, where alignment columns are resampled, AliFilter randomly resamples alignment rows (i.e. sequences). This way, multiple sets of features are computed for each alignment column, which can be separately assessed by the model; given the number of bootstrap replicates *b* and the bootstrap threshold $t_{b}$, a column is preserved only if at least $t_{b}\cdot b$ replicates have a score p>t. Different values for *b* and $t_{b}$ are assessed during model validation, in order to evaluate their effect on the target metric *M* (i.e. *MCC*, *A*, or $F_{\beta }$). However, to account for the additional computational effort involved in computing bootstrap replicates, a regularized score, S=M−b⋅E is used to determine the best value of *b*, where *E* is a penalizing constant (by default, $5\cdot 10^{-5}$). This ensures that a further 100 bootstrap replicates are only performed if they improve the prediction score by at least 0.005.

In the model testing step, AliFilter applies the validated model to the test dataset, without modifying any parameter values. It then compares the results with the input mask and computes the value for multiple performance metrics, including the *MCC*, *A*, $F_{\beta }$, *C*, the receiver operating characteristic (ROC) curve (Peterson et al. 1954; Junge and Dettori 2018), the area under the curve (AUC), and more. These can be used to collectively determine whether the model performance is satisfactory, i.e. if it is a good approximation of the manual filtering approach.

## Methods

### Datasets

During the development of AliFilter, eight datasets were used to train the software and assess its performance, including a wide variety of taxonomic groups and multiple types of sequence data (Table 2). A total of 451 alignments (982,813 columns) were analyzed, consisting of 273 amino acid sequence alignments (232,791 columns), 120 DNA alignments (461,610 columns), and 58 ribosomal rRNA (rRNA) alignments (288,412 columns). Datasets 1 to 4 consist of amino acid sequences from various groups of bacteria and archaea, Datasets 5 to 7 consist of DNA sequences of protein-coding genes from various groups of hexapods, and Dataset 8 consists of small subunit (SSU) and large subunit (LSU) sequences from bacteria, archaea, and eukaryotes. Datasets 1, 4, 5, 6, and 7 originate from studies that have been previously published (Moody et al. 2022; Cicconardi et al. 2023; Bianchini et al. 2024) or are in preparation (Supplementary Data), while the other datasets were generated for this study; all datasets are further described in the Supplementary Data and are available in the AliFilter GitHub repository.

**Table 2:** **Datasets used during AliFilter development.** Eight primary datasets (1-8) were used to analyze the performance of AliFilter and to train alignment filtering models. Three secondary datasets (9-11) were generated by merging the primary datasets. For each dataset, the table includes the number of alignments in the training, validation, and test set, as well as the total number of alignment columns.

	Dataset name	Type	Alignments (Columns)		
			Training	Validation	Test
**1**	Cyanobacteria (phylogenomic)	AA	…	…	139 (82,113)
**2**	Cyanobacteria (BUSCO)	AA	20 (36,176)	10 (13,256)	10 (12,386)
**3**	Rhodobacteraceae (BUSCO)	AA	20 (8,886)	10 (4,314)	10 (3,856)
**4**	Prokaryotes (COG)	AA	28 (37,910)	13 (17,045)	13 (16,849)
**5**	Collembola (BUSCO)	DNA	20 (62,899)	10 (36,893)	10 (37,928)
**6**	Formicidae (BUSCO)	DNA	20 (72,988)	10 (44,019)	10 (45,085)
**7**	Heliconiini (scOG)	DNA	20 (84,599)	10 (41,989)	10 (35,210)
**8**	rRNA (SILVA)	RNA	30 (148,675)	14 (73,158)	14 (66,579)
**9**	Full dataset (#1-8)	Mixed	158 (452,133)	77 (230,674)	216 (300,006)
**10**	Prokaryotes (Proteins) (#2-4)	AA	68 (82,972)	33 (34,615)	33 (33,091)
**11**	Hexapoda (DNA) (#5-7)	DNA	60 (220,486)	30 (122,901)	30 (118,223)
**12**	Animals (Mitochondria)	Mixed	…	…	35 (19,435)
**13**	*Caudoviricetes* (VOG)	AA	…	…	73 (141,007)

Three more datasets were generated by merging the eight primary datasets: Dataset 9 consists of all the alignments in Datasets 1 to 8, Dataset 10 corresponds to Datasets 2 to 4, and Dataset 11 originates from Datasets 5 to 7. To test the AliFilter model against other types of data that were not used during its development, we additionally generated Dataset 12 (from 35 alignments of animal mitochondrial sequences) and Dataset 13 (consisting of 73 alignments of virus orthologous groups (Trgovec-Greif et al. 2024) from the class *Caudoviricetes*); for more information, see Supplementary Data. With the exception of Datasets 1, 12, and 13, the alignments from each dataset were partitioned into a training set, a test set, and a validation set, following proportions of approximately 2:1:1 (Table 2). For the remaining datasets (1, 12, and 13), the alignments were used exclusively for model testing. For benchmarking purposes, we also used the SSU and LSU Ref NR99 alignments (across all domains of cellular life) from release 138.2 of the SILVA database (Quast et al. 2013), as well as alignments of the (bacterial) bac120 markers from release 226 of the Genome Taxonomy Database (GTDB) (Parks et al. 2022) (Supplementary Data). Finally, an additional dataset containing 172 single-copy protein coding genes from 39 animal species (Yu et al. 2024), as well as a set of 117 simulated alignments from 1398 “taxa,” were used to investigate the effect of different filtering strategies on phylogenetic tree reconstruction. These alignments were not manually filtered and were not used for model training or testing.

### Sequence alignment, filtering, and phylogenetic analyses

All alignments were built employing MAFFT (Katoh and Standley 2013) with the –localpair –maxiterate 1,000 options, and each alignment was manually filtered using the online AlignmentViewer utility (Bianchini 2017). This involved first removing columns with a gap content greater than 85%, and further refining the alignments by visually identifying and removing misaligned or noisy positions. To ensure consistency in the datasets, all alignments were filtered by the same person (GB). To determine whether different manual filtering approaches produce appreciably different results, Datasets 10 and 11 were also manually filtered by ERRM and FC, respectively, based on their own experience in analyzing sequence alignments. This approach used a less stringent gap content threshold of 95%, followed by visual identification and removal of misaligned or noisy positions. Datasets filtered by ERRM and FC were compared with the same datasets filtered by GB using the previously described approach (85% gap threshold followed by manual refinement).

Manual filtering and AliFilter results across all datasets were also compared with alignments filtered using five existing tools: BMGE v2.0 with default settings (Criscuolo and Gribaldo 2010); trimAl v1.4.1 with the “automated1” preset (Capella-Gutiérrez et al. 2009); Gblocks v0.91b (Castresana 2000) with the “b1” and “b2” parameters set to 0.5n(where *n* is the number of sequences in the alignment), the “b3” parameter set to 1, “b4” set to 6, and “b5” to h; Noisy v1.5.12 with default settings (Dress et al. 2008); and ClipKIT v1.4.1 with default settings (Steenwyk et al. 2020). To assess the effect of alignment filtering on a phylogenomic analysis, filtered alignments from Dataset 1 and from the animal dataset were concatenated, and a phylogeny was estimated using IQ-TREE v2.3.6 (Minh et al. 2020) under the Q.pfam_gb+C20+R7 model (Si Quang et al. 2008; Le et al. 2012; Minh et al. 2021), with 1,000 ultrafast bootstrap replicates (Hoang et al. 2018). Additionally, a simulated dataset was generated using the reference tree of 1,398 representative Gemmatimonadota from the GTDB (Parks et al. 2022) as a template; 117 alignments were created using IQ-TREE's –alisim option (Ly-Trong et al. 2022) to “mimic” real alignments of phylogenetic markers using the LG+R8 model; these were realigned and filtered, and trees were built in a partitioned analysis (-p option) also employing the LG+R8 model (Supplementary Data). Three replicate analyses were run for each tool; the execution time, amount of RAM required, and total length of the maximum-likelihood tree were recorded for each analysis of Dataset 1 (Table S2) and compared with the results obtained for unfiltered alignments, alignments filtered using AliFilter, and alignments filtered using other tools.

To assess the effect of alignment filtering on the results of phylogenetic estimation, we created tree space plots based on the Robinson-Foulds distance ((Robinson and Foulds 1981), which assesses the tree topology without considering branch lengths, and on the Frobenius distance computed on patristic distance matrices (which assesses the overall tree shape, see Supplementary Data for more details). To reduce computation time, the 3,000 ultrafast bootstrap replicates obtained for each tool were subsampled, preserving only 300 representatives per tool. Classical multidimensional scaling (Torgerson 1952) was used to convert tree distances to coordinates in a 2D space (Fig. 5b, Figures S1c, S2c, S7, S9b, c and S11). Phylogenetic trees were plotted using TreeViewer v2.2.0 (Bianchini and Sánchez-Baracaldo 2024).

### AliFilter models

The main AliFilter model, which is included as the default model in the program, was developed using the alignments in Dataset 9 (full dataset). Two additional models were developed using Dataset 10 and Dataset 11 as filtered by GB, and alternative versions of these models were developed using Dataset 10 filtered by ERRM and Dataset 11 filtered by FC. To evaluate the differences between these approaches, models trained and validated using GB's datasets were tested against ERRM's and FC's test datasets, and vice versa. Furthermore, AliFilter's performance when replicating other tools was assessed by training and testing models with alignments from Dataset 9 filtered using BMGE, ClipKIT, Gblocks, Noisy, and trimAl.

To analyze the effect of incorrect column assignment in the input data on the trained models, we used AliFilter's “–mistakes” command line option to simulate up to 50% random errors in the input mask for the training and validation datasets, with five replicates for each tested proportion of mistakes. The models were then tested against the unmodified test dataset (which we assume contains a negligible number of mistakes). Furthermore, to determine the effect of varying amounts of training and validation data on the model, we trained and validated models on randomly selected small subsets of alignments from Dataset 9. Each model was then tested against the full test dataset, with ten replicates for each data point.

### Implementation

AliFilter is written using the C# programming language, running under the open-source.NET 8 runtime (Microsoft 2021). Executables are available for Windows, Linux, and macOS operating systems, both for x64 processors (e.g. Intel or AMD processors) and for ARM processors (e.g. Apple Silicon or Qualcomm Snapdragon). AliFilter is free and open-source software, released under the GNU GPLv3 license (Free Software Foundation 2007); in addition to the base.NET runtime libraries, the program uses the Accord.NET machine learning framework (Souza et al. 2017) to train models, the Math.NET Numerics library (Math.NET Team 2009) for statistical analyses, and the VectSharp library (Bianchini 2019) to produce graphics.

AliFilter models are stored in JSON-formatted text files, which allows them to be easily imported in other software and programming languages. Combined with the relative simplicity of the logistic model, this means that AliFilter models can be readily combined with other tools. As a proof of concept, we provide implementations for AliFilter filtering in C, R, Python, and JavaScript in the AliFilter repository, and a practical implementation of the JavaScript code is available in the online AlignmentViewer utility (Bianchini 2017).

## Results

### Model performance

In our analyses, we evaluated the performance of AliFilter models using two key metrics: the accuracy (*A*) and the Matthews correlation coefficient (*MCC*) (Matthews 1975). *A* (ranging from 0 to 1) represents the proportion of alignment columns for which the model and the “true” mask agree, while *MCC* (ranging from −1 to 1) is a measure of the correlation between model assignments and “true” assignments; since our test datasets are not unbalanced, both measures should provide similar interpretations (Chicco and Jurman 2020). We also considered the model confidence score (*C*), which provides a measure of the average confidence of the model assignments, although its value does not depend on how many correct or incorrect assignments have been made.

**Figure 2:**
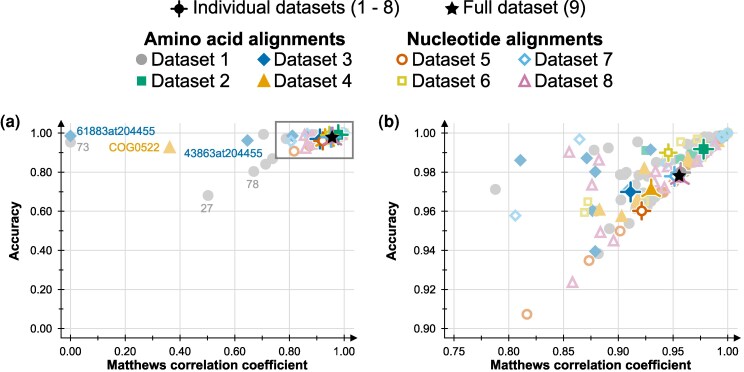
Performance metrics for the main AliFilter model. (a) Each data point in the plot represents one of 216 alignments, while crosshair symbols represent the overall results for the eight datasets. The black star symbol represents the overall result for the complete dataset (Dataset 9). Alignments with Matthews correlation coefficient <0.70 are highlighted and mentioned in the text. The grey square identifies the area shown in more detail in part b. (b) Zoomed-in view of the datapoints with Accuracy ≥0.90 and Matthew’s correlation coefficient ≥0.75. Dataset numbers as in Table 2.

The AliFilter model trained on the full dataset (Dataset 9) shows excellent performance across all metrics (Fig. 2), both when compared to the full test dataset (A=0.98, MCC=0.96, and C=0.94), as well as when compared to the test data for individual datasets (Table S1). Strong performance metrics are also observed when testing the model against single alignments, with very few exceptions (Fig. 2, Table S1). These outliers include alignments 73 (Dataset 1), 61,883 at 204,455 and 43,863 at 204,455 (Dataset 3), and COG0522 (Dataset 4), which display low *MCC* but high *A*: this is because in the manually filtered alignments, a very high proportion of columns (100%, 98.5%, 93%, and 94%, respectively) is preserved for these four alignments; therefore, even a small number of false negatives skews the *MCC* score, without significantly affecting the accuracy. On the contrary, alignments 27 and 78 (Dataset 1) represent proteins with a conserved domain shared across all taxa, as well as other domains that are only present in some representatives (Figure S1a and S2a). The model tends to discard most of these domains, while the manual filtering approach preserves them, and as a result, these have low values for both *MCC* and *A*; however, the ROC curves (Figure S1b and S2b) suggest that AliFilter can still correctly rank most columns in these alignments, and thus, higher *MCC* and *A* could be produced by using different thresholds. When comparing gene trees obtained with these alignments, for alignment 27, manual filtering and AliFilter produce different gene trees (Figure S1c), while for alignment 78, the resulting trees are very similar (Figure S2c); however, in both cases, the gene trees are largely different from the species tree inferred using the full dataset, as is often the case for single-gene bacterial phylogenies (Dagan and Martin 2007; Moody et al. 2022; Williams et al. 2024). When tested against novel datasets excluded from its development, the model produced similarly good results (A=0.98, MCC=0.96, C=0.90 on Dataset 12, and A=0.98, MCC=0.93, C=0.85 on Dataset 13, Table S1, Figure S3).

**Figure 3:**
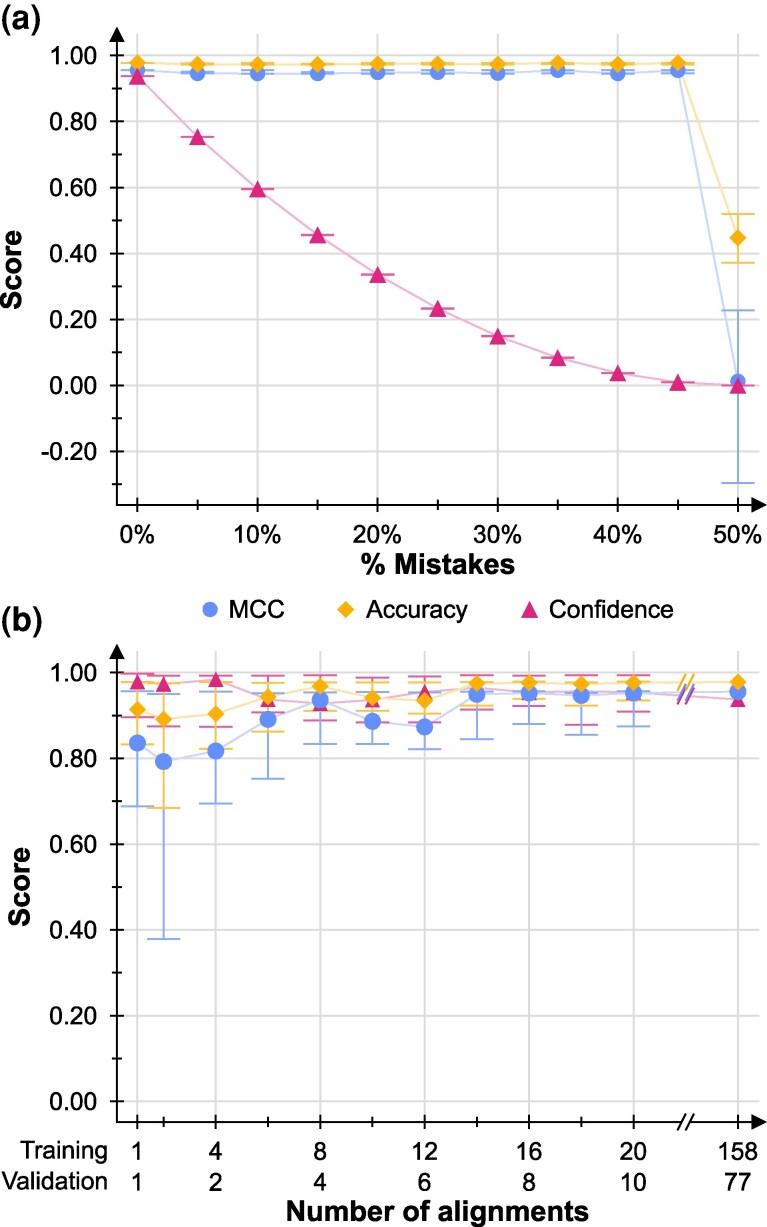
Effect of mistakes and training dataset size on model performance. (a) Mistakes. Performance metrics are shown as a function of the proportion of mistakes introduced in the training and validation dataset. For each tested proportion of mistakes, n=5 replicates were performed; the symbols show median values, while the whiskers span the range of observed values. (b) Training dataset size. Performance metrics as a function of the size of the training and validation datasets. For each dataset size, n=10 replicates were performed; the symbols show median values, while the whiskers span the range of observed values. *MCC*: Matthew’s Correlation Coefficient.

Manually filtering sequence alignments is laborious; as such, inaccuracies resulting from human error may creep into datasets used to train AliFilter models. To analyze their effect, we trained and validated models with increasing proportions of random artificial mistakes in the data (Fig. 3a), showing that the *A* and *MCC* of these models are largely unaffected until the proportion of mistakes reaches 50%. Instead, model confidence (*C*) is clearly affected by the presence of incorrect assignments in the training and validation data, dropping from 0.94 to 0.75 when the dataset contains a small number of mistakes (5%, Figure S4); however, it is important to note that this does not affect the performance of the model in terms of which columns are preserved or discarded. Another crucial point when creating a machine learning model is the use of sufficient training data to capture the variation of the unknown input data on which the model will be applied. For the full AliFilter model, we included 235 alignments for training and validation, but in a real-world application, it is likely that a user may have fewer datasets at their disposal. To determine how many alignments are necessary to train an effective AliFilter model, we trained and validated models using randomly selected subsets of Dataset 9, before testing them against the full test set (Fig. 3b); this showed that scores comparable to the full model can be obtained with as little as 14 alignments for the training set and 7 for the validation set.

Even with vast datasets, training AliFilter models is computationally efficient and can be parallelized effectively, depending on the number of available CPUs. To demonstrate this, we benchmarked the amount of time required for feature computation and model training, validation, and testing, using three separate machines that are representative of both relatively old and more recent hardware (Figure S5). Under the optimal parallelization strategy, generating the complete AliFilter model (including training, validation, and testing) took 1 h and 40 min on a 6-year-old laptop, while on a 4-year-old high-end desktop, this took around 26 min. For a more realistic estimate for end-users, creating a model using only Dataset 4 required around 4 min on the laptop and just over 1 min on the desktop. Although real-life performance will depend on various factors (e.g. the number of logical processors and CPUs, RAM speed, and I/O latency), these results show that the most time-consuming step will generally be the manual filtering of alignments in order to prepare the training dataset.

### Comparisons with other tools

**Figure 4:**
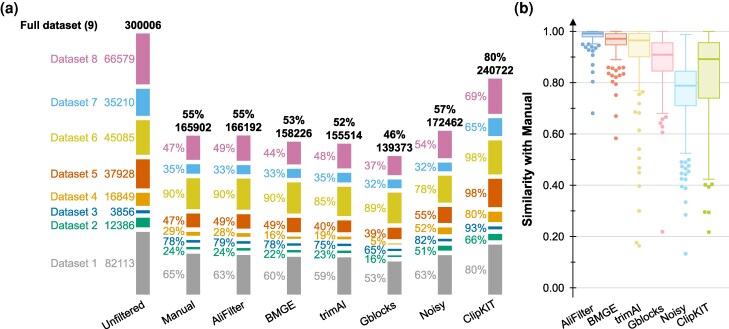
Comparison between alignment filtering tools. (a) Proportion of preserved columns. The total number of columns in the unfiltered datasets is shown, together with the proportion of columns preserved by each alignment filtering approach. (b) Comparison between manual filtering and other filtering approaches. The box plots show the similarity between alignments filtered using each tool and the manually filtered alignments. Whiskers represent 1.5 interquartile ranges above and below the median. Outliers are shown as swarm plots. Note that low similarity scores do not indicate low performance, since filtering tools other than AliFilter do not aim to replicate manual filtering. Dataset numbers as in Table 2.

We evaluated the differences between various filtering approaches by comparing test set alignments that had been filtered manually, using AliFilter, or using other software tools (Fig. 4). The unfiltered test dataset contained a total of 300,006 alignment columns, 55% of which are preserved via manual filtering (Fig. 4a); AliFilter achieves highly similar results across all datasets. Compared to this, BMGE (Criscuolo and Gribaldo 2010), trimAl (Capella-Gutiérrez et al. 2009), and Gblocks (Castresana 2000) preserve fewer columns, while Noisy (Dress et al. 2008) and ClipKIT (Steenwyk et al. 2020) preserve more. This highlights how each tool targets a different balance between discarding noisy data and preserving as much information as possible. To further compare alignments filtered by these tools, we computed similarity scores across manual and tool-based filtering of alignments (i.e. the proportion of columns that both manual filtering and each tool agree on preserving or deleting, Fig. 4b). It should be noted that a low score in this context does not mean that the tool performs poorly, but rather that the alignments it produces are notably different from the manually filtered ones. As expected, alignments filtered by AliFilter are the most similar to the manual filtering approach and, while filtering with other tools can occasionally produce similar results, there is a high degree of variation in the accuracy scores, with a minimum of A=0.13.

To assess our software's ability to quickly process large amounts of data across different taxonomic groups, we used AliFilter and other tools to filter the inter-domain SSU (510,495 sequences and 50,000 residues) and LSU (95,279 sequences and 149,999 residues) Ref NR99 alignments from the SILVA database (Quast et al. 2013), as well as full alignments of the bacterial bac120 markers (each containing on average more than 100,000 sequences and 15,000 columns) from the GTDB database (Parks et al. 2022). AliFilter was consistently faster than other tools, and even the largest alignment was filtered in less than 10 min (Figure S6a); RAM usage was similar between AliFilter and BMGE for alignments up to 5GiB in size, with AliFilter requiring more memory beyond this threshold, although BMGE crashed when analyzing the largest alignment (Figure S6b). Consistent with the results shown in Fig. 4, AliFilter generally preserved a number of columns similar to the median length of unaligned sequences, while alignments filtered by BMGE and Gblocks were shorter (or even empty), and ClipKIT preserved many more columns (Figure S6c). These results show that AliFilter is well-suited to analyze large datasets, provided that the amount of available memory is commensurate with the alignment size.

**Figure 5:**
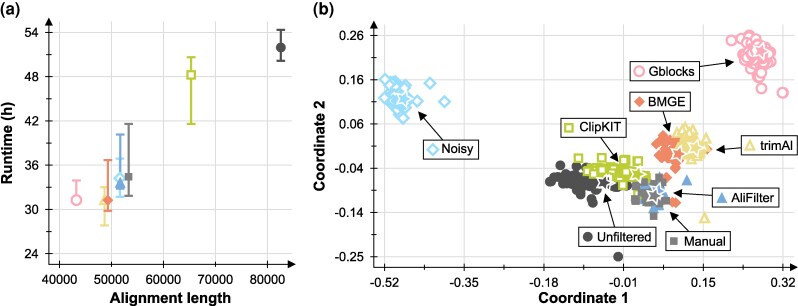
Effect of alignment filtering on a phylogenomic-level analysis. (a) Relationship between filtered alignment length and total runtime. For each tool, a bar shows the range of runtimes observed in three independent replicate analyses in IQ-TREE 2.3.6 (Minh et al. 2020); symbols indicate the median runtime. (b) Visualization of the tree space according to the Frobenius distance. Each symbol on the plot represents a single ultrafast bootstrap replicate tree (300 for each tool) (Hoang et al. 2018). Stars represent the maximum-likelihood trees (three for each tool, often overlapping).

We next sought to verify whether different alignment filtering strategies would have a practical effect in a phylogenetic analysis. To do this, we used the 139 alignments in Dataset 1 for a concatenated phylogenomic analysis of Cyanobacteria in IQ-TREE (Minh et al. 2020); we then compared software runtime and memory requirements, as well as the final tree topology, when using alignments filtered with different tools. As expected, longer alignments result in longer execution times for phylogenetic inference (Fig. 5a, Table S2); in particular, the AliFilter dataset reduced execution time by around 35% compared to the unfiltered dataset. Noisy produced a similar result, while BMGE, trimAl, and Gblocks all had slightly shorter runtimes. Instead, the ClipKIT dataset had a similar runtime as the unfiltered dataset, as this tool preserved 80% of the alignment columns in our analysis (Fig. 4a).

Identical tree topologies were obtained when using unfiltered alignments and alignments filtered with ClipKIT and AliFilter (Figure S7); two of the three replicates filtered with Noisy also produced the same topology, while BMGE, trimAl, and Gblocks produced different results. The Gblocks replicates, in particular, produced three distinct trees, which suggests that not enough phylogenetic signal was preserved to confidently resolve a single topology. Most of the differences in tree topology across the various tools were found within the order Nostocales (Figure S8); when comparing these results to previously published data (Strunecký et al. 2023), we noticed that while the unfiltered alignment, manual filtering, AliFilter, ClipKIT, and Noisy recovered the family Nodulariaceae as monophyletic, and BMGE, trimAl, and Gblocks rendered it polyphyletic. However, in all cases, the family Leptobasaceae was nested within the family Nostocaceae, likely because Dataset 1 only includes a single strain from this family.

We next used the Frobenius distance between normalized trees (Supplementary Data) to assess the overall similarity in the tree “shape”, i.e. the relative lengths of the tree branches. AliFilter and ClipKIT produce overall similar trees to the unfiltered alignments (Fig. 5b), while trees built using BMGE and trimAl are more dissimilar. Gblocks and Noisy present the highest degree of dissimilarity to the full alignment; for Noisy, this contrasts with the topology-only comparisons (Figure S7) and suggests that the preserved columns provide biased estimates for branch lengths, although they recover the same phylogenetic relationships as the full alignment.

In a phylogenetic analysis including 172 single-copy protein-coding genes from 39 animal species, identical topologies were produced by the unfiltered alignments, AliFilter, BMGE, ClipKIT, trimAl, and Noisy for the phylum Chordata (Figure S9, S10), in agreement with previous results (Yu et al. 2024). Outside of this phylum, however, only Noisy recovered (with low support) a monophyletic Deuterostomia clade containing Chordata and Ambulacraria, while the other tools weakly supported the “Centroneuralia” clade instead, thus grouping together chordates and protostomes to the exclusion of Ambulacraria (Kapli et al. 2021). A monophyletic Deuterostomia clade was only found with high support when using alignments filtered by Gblocks (Figure S10); however, this also resulted in very uncertain relationships within chordates because this approach discards 97% of the alignment data (Figure S9a). This dataset, which only contains eight non-chordate species, is likely not adequate to fully address this question, but these results agree with those of recent studies, suggesting that strong support for deuterostome monophyly may arise as the result of systematic errors (Kapli et al. 2021; Serra Silva et al. 2025).

Finally, we tested the effect of alignment filtering on a simulated dataset, which allows us to compare the inferred phylogenetic tree with a known “true” underlying tree. However, simulated sequence datasets have been shown to be limited with respect to their real-world implications (Trost et al. 2024). In this analysis, the unfiltered alignments and the alignments filtered with all the tools we tested produced identical maximum-likelihood tree topologies, with very high support (for all nodes, UFBoot ≥ 97% for Gblocks, ≥ 99% for BMGE, and 100% for the other tools). The trees were also very similar in terms of relative branch lengths, with the most discernible difference between the different filtering strategies being the overall scale of the tree (Figure S11c), as each tool preserves a different proportion of slow- and fast-evolving sites (this was also observed for the Cyanobacteria dataset, Table S2).

### Comparison between AliFilter models

To determine the effect of human influence and to verify AliFilter's efficacy in emulating alternative filtering approaches, we trained models using alignments filtered by different authors (GB, ERRM, and FC). Alignments in Dataset 10 were independently filtered by GB and ERRM, who preserved 32% and 33% (respectively) of the columns in the test set. Comparing the two filtered test sets, as well as the models trained on this dataset (Table S3), showed that the filtering approaches used by GB and ERRM are highly similar (A=0.95, MCC=0.90), and both can be replicated faithfully by AliFilter (A=0.98 and MCC=0.97 for the model trained and tested on GB's data; A=0.95 and MCC=0.89 for the model trained and tested on ERRM's data; A=0.95 and MCC=0.90 for GB's model tested on ERRM's data; A=0.98 and MCC=0.95 for ERRM's model tested on GB's data).

On the other hand, there is a greater discrepancy between the filtering strategies used by GB and FC, which preserved 60% and 45%, respectively, of the test set for Dataset 11 (A=0.85, MCC=0.73). This is also reflected in the models trained on these alignments (Table S3), which perform well when compared to the test set filtered by the same person, but more poorly against the test set filtered by the other (A=0.98 and MCC=0.95 for the model trained and tested on GB's data; A=0.98 and MCC=0.95 for the model trained and tested on FC's data; A=0.85 and MCC=0.74 for GB's model tested on FC's data; A=0.83 and MCC=0.71 for FC's model tested on GB's data). These results highlight that AliFilter can accurately replicate both GB's and FC's approaches in their distinctiveness. Further comparisons between a model trained on one dataset and tested against a different dataset also show good performance metrics when both the training/validation, and the test sets have been filtered by the same person (A≥0.94, MCC≥0.87, Table S4).

Although the main goal of AliFilter is to automate manual filtering, any filtered alignments can, in principle, be used to train AliFilter models. To test this, we created models that emulate the filtering approach of other existing tools (Table S5), showing that AliFilter can accurately replicate the filtering approach of Gblocks and BMGE, but not ClipKIT, Noisy, or trimAl, likely due to the different alignment features used by these tools.

## Discussion

Our results show that AliFilter can be used to accurately automate the manual alignment filtering approach of different individuals, by producing personalized models that replicate each approach, with a relatively small amount of data required to produce a reliable model. Processing alignments with AliFilter produces deterministic and reproducible results, unlike manual filtering, which can introduce inaccuracies and biases in the dataset due to human error. The model training process is resilient to such mistakes, showing practically no reduction in performance as long as the mistakes do not affect the majority of the training data.

When provided with conflicting alignments, AliFilter models extract and reproduce the filtering criterion observed in the majority of the training data, rather than taking an average of multiple different criteria. If the training data consists of a range of alignments filtered with multiple approaches (e.g. by different individuals or tools), the resulting model will represent the modal approach (i.e. the person who filtered the most alignments). Thus, if a consensus filtering approach across different styles is desired, the appropriate procedure would be to use a different model for each filtering style and then compare the resulting alignment masks. AliFilter's –bitwise/-b command-line option can be used for this.

A previous machine learning approach to manual filtering of amino acid alignments, MANUEL (Blouin et al. 2009), performed this task using a support vector machine (SVM) classifier with a non-linear kernel. This software was implemented as an online web server where users could register to filter alignments and contribute to the training dataset. In contrast, AliFilter uses a more efficient logistic model and consists of a single executable file able to be run locally on the end-user's computer (or cluster) without online dependencies, allowing for integration into existing pipelines, for both amino acid and nucleotide alignments.

A further difference between AliFilter and MANUEL lies in the features used by the two programs. The four features used by MANUEL include the proportion of gaps and three metrics computed using a neighbor-joining (NJ) tree (Saitou and Nei 1987) inferred from the unfiltered alignment: the “normalized site likelihood ratio,” which compares the likelihood of each column under the NJ tree and a star tree; the “SR,” representing the relative evolutionary rate of the column; and the “normalized parsimony count,” which estimates the parsimoniousness of the pattern of gap and non-gap residues. In contrast, AliFilter does not use any features that require inferring a phylogenetic tree, because these likely do not directly influence the decision to preserve or delete a column by a human annotator. Furthermore, MANUEL considers the “neighborhood” of each column by including the features of adjacent columns in its classifier model; instead, AliFilter uses a simpler approach, where only the proportion of gaps affects neighboring columns. Comparing results obtained with AliFilter and MANUEL is unfortunately not possible because both the MANUEL webserver and the source code to the software are no longer available (C. Blouin, personal communication).

The large number of alignment filtering tools in active use (Castresana 2000; Dress et al. 2008; Capella-Gutiérrez et al. 2009; Criscuolo and Gribaldo 2010; Steenwyk et al. 2020) indicates that there is no consensus filtering approach. AliFilter includes the model trained on the full dataset (Dataset 9) as the default if no model is specified, but this represents just one possible filtering technique and is unlikely to be appropriate in all cases. Users are thus encouraged to use our software to train their own filtering models for their analyses. These could include user-specific models (where the model is trained on multiple datasets analyzed by the same user), dataset-specific models (where training occurs on a single dataset filtered by multiple users), or user-specific and dataset-specific models (where the input data comes from a single dataset filtered by the same user).

Training a machine-learning model can appear a daunting task, and special care should be taken to ensure that the trained model produces good results when presented with new data (Bishop 2006). To simplify this process and make interpretation easier, AliFilter produces validation, filtering, and testing reports. These reports (Supplementary Data) contain analyses of the input data, as well as several plots and statistics to provide insight into the model training process and its results. They also include clear definitions and interpretation guidelines for the results they present, aimed at allowing even users without machine-learning expertise to understand the overall message. When using a custom AliFilter model for an analysis, we recommend users include the validation and test reports for the model together with the data they used, as these will improve the interpretability and reproducibility of the analysis.

When applying machine learning approaches to biological data, it is important to be aware of potential pitfalls that may cause an overestimation of the model's performance if information “leaks” from the test set into the training and validation sets (Whalen et al. 2022). AliFilter avoids this by treating each sequence alignment as an unbreakable unit when performing data partitioning, to ensure that columns from the same alignment will not be used for both training and evaluation purposes; using alignment data from a variety of gene and protein families can also help guard against this issue.

When comparing AliFilter (and manual filtering) results with those obtained with other tools, we showed that there are both similarities and differences. AliFilter can create models replicating BMGE and Gblocks, but not ClipKIT, trimAl, or Noisy (Table S4). This suggests that, while the filtering criteria used by the first two tools can be accurately represented by a logistic model, the other tools’ criteria cannot. BMGE mainly uses an “entropy” measure to determine whether to preserve or delete alignment columns (Criscuolo and Gribaldo 2010) and Gblocks uses a combination of percent identity and proportion of gaps (Castresana 2000). These three metrics are included within the six features used by AliFilter (Table 1), which likely explains why our software is able to replicate BMGE's and Gblocks's filtering approach. On the other hand, ClipKIT's strategy is based on parsimony-informativeness (Steenwyk et al. 2020) and Noisy analyses the distribution of characters along cyclically ordered taxa (Dress et al. 2008), both of which are likely not adequately represented by AliFilter's six features. Interestingly, trimAl also uses sequence identity and gap proportion to analyze alignment columns (Capella-Gutiérrez et al. 2009); however, for the “automated1” setting we tested, the program used conservation thresholds that vary for each alignment, which may explain why AliFilter can emulate it relatively poorly.

On a concatenated dataset of 139 cyanobacterial genes, alignments filtered with AliFilter produce similar estimates to full alignments (Fig. 5b, Figure S7), while being significantly shorter and thus requiring less computation time and system memory (Fig. 5a, Table S2). While the other tools we tested can also produce similar phylogenies (ClipKIT) or short runtimes (BMGE, trimAl, and Gblocks), none of them can achieve both goals at the same time. Similar results were observed on a dataset of 172 animal genes, although in this case, BMGE also produced similar results to AliFilter, ClipKIT, and the unfiltered alignment (Figure S9). Interestingly, alignments filtered with each tool produce different estimates for the total length of the tree (Table S2, Figures S8, Figure S10). This suggests that each tool preserves a different “blend” of fast- and slow-evolving sites; while Noisy and ClipKIT preserve alignment columns regardless of their rate of evolution, AliFilter, BMGE, trimAl, and especially Gblocks are more likely to preserve columns with low evolutionary rates, which may or may not be desirable for a specific analysis (Brinkmann et al. 2005; Duchêne et al. 2022; Superson and Battistuzzi 2022; Rangel and Fournier 2023).

Whether alignment filtering provides an improvement in accuracy for phylogenetic inference or whether it would be equivalent to use raw, unfiltered alignments is a matter of debate (Talavera and Castresana 2007; Tan et al. 2015). For example, in the animal dataset, the estimated tree topology was largely unaffected by the choice of filtering strategy (Figure S9, Figure S10), with the few observed differences affecting a single low-support node; similarly, all filtering approaches produced essentially identical trees on a simulated dataset (Figure S11). However, an undeniable consequence of alignment filtering is that subsequent phylogenetic analyses will use a reduced amount of sequence data. As long as the filtering approach does not remove an excessive amount of phylogenetically relevant data, thus negatively impacting the analysis (Talavera and Castresana 2007), this provides a net benefit, in the form of reduced computation time and, hence, reduced energy consumption. For example, on our system, the lower RAM usage and total ∼50 h reduction in computation time (across three replicates) observed on the concatenated phylogenomic dataset of Cyanobacteria reduced energy consumption by 36%, saving 37.5 kWh of electricity and 8.66 kg of CO_2_ emissions (Lannelongue et al. 2021). As we head into a climate crisis (Ripple et al. 2020) and emissions and energy consumption by scientific analyses become a growing concern (Wu et al. 2022), alignment filtering is one step (of many) toward greener computing (Paul et al. 2023).

## Conclusion

AliFilter is a free and open-source software, released under the GPLv3 license. The source codes, as well as compiled native binaries for computers running x64 and ARM versions of Windows, Linux, and macOS, are available from the software's GitHub repository at https://github.com/arklumpus/AliFilter. The GitHub repository also contains documentation and extensive tutorials to help users. Example implementations are also provided to aid other developers to include the option to use AliFilter models within other software. AliFilter's codebase is designed with expandability in mind, and its object-oriented approach means that it will be relatively easy to extend the software to support additional alignment features, or even machine-learning models other than the currently implemented logistic model. According to our tests, this was not necessary to automate manual alignment filtering, but, in the future, AliFilter may be able to approach more complex filtering problems, with applications, for example, in the fields of sequence domain recognition or protein/RNA secondary and tertiary structure analysis.

## Supplementary Material

msag097_Supplementary_Data

## Data Availability

The source code for AliFilter, as well as all sequence alignments that were analysed, are available from GitHub at https://github.com/arklumpus/AliFilter and archived on Zenodo under DOI 10.5281/zenodo.14861812.
