# Supplementary material for: AliFilter: a machine learning approach to alignment filtering
Source: Mol Biol Evol. 2026 Apr 10;43(4):msag097. doi: 10.1093/molbev/msag097 (PMC13108598; doi:10.1093/molbev/msag097)
Supplement: msag097_Supplementary_Data [file msag097_supplementary_data.zip › alifilter.test_report.pdf]

Model test report

Created by AliFilter version 1.0.0 on 24th Nov, 2024 at 18:55.

MD5 checksum of the model this report refers to: `E95D52E3F38227FDA87BC60EBBE59E34` (feature signature: `8D2F81A3625201DA548E08978D516E0C`)

This report may be included in any analysis using this model.

This report is machine-readable. If the report file is called `report.pdf`, you can export the tested model to a file called `model.json` by running:

- On a Unix machine:

```
grep -a "@model" report.pdf | sed "s/@model//g" > model.json
```

- On a Windows machine (within a PowerShell environment):

```
findstr "@model" report.pdf | %{$_ -replace "@model",""} > model.json
```

You can also export the performance metric summary table by running:

- On a Unix machine:

```
grep -a "@metric" report.pdf | sed "s/@metric//g"
```

- On a Windows machine (within a PowerShell environment):

```
findstr "@metric" report.pdf | %{$_ -replace "@metric",""}
```

Test data analysis

The model was tested using data from 300006 alignment columns, of which 165904 (55.30%) were preserved and 134102 (44.70%) were deleted (**Fig. 1**). For each alignment column, 6 features were computed (**Table 1**), which were analysed in a Principal Component Analysis (PCA) and in a Linear Discriminant Analysis (LDA).

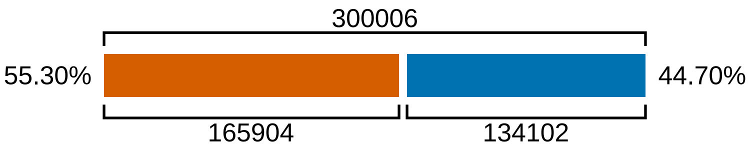

Figure 1. Proportion of preserved columns. The figure shows the proportion of data columns that were preserved (in orange, on the left) or deleted (in blue, on the right).

Table 1. Alignment features. Features that have been computed for each alignment column, including a brief description and the observed range, mean and standard deviation (SD) for each of them.

Name	Description	Values
Gap proportion	Proportion of sequences that have a gap in the column.	*Range*: 0 - 0.9997<br>*Mean*: 0.4547<br>*SD*: 0.4655
Percent identity	Frequency of the most common residue in the alignment column, excluding gaps.	*Range*: 0.0003 - 1<br>*Mean*: 0.4505<br>*SD*: 0.4182
Distance from extremity	Number of residues between the column and the closest extremity (start or end) of the alignment.	*Range*: 0 - 12552<br>*Mean*: 1583.1164<br>*SD*: 2330.7693
Entropy	Shannon entropy for the residue frequencies in the column, excluding gaps.	*Range*: 0 - 2.8354<br>*Mean*: 0.4179<br>*SD*: 0.5533
Gap proportion (±1)	Average of the proportion of gaps between the column, 1 preceding column(s), and 1 subsequent columns.	*Range*: 0 - 0.9997<br>*Mean*: 0.4547<br>*SD*: 0.4468
Gap proportion (±2)	Average of the proportion of gaps between the column, 2 preceding column(s), and 2 subsequent columns.	*Range*: 0 - 0.9997<br>*Mean*: 0.4547<br>*SD*: 0.4388

A PCA (**Fig. 2**) uses a linear transformation to transform the data to a coordinate system where each coordinate (component) explains as much of the variance of the data as possible, while being orthogonal to the previous components. This is useful to show the distribution of the input data, but a PCA, on its own, cannot be used to decide whether an alignment column should be preserved or not.

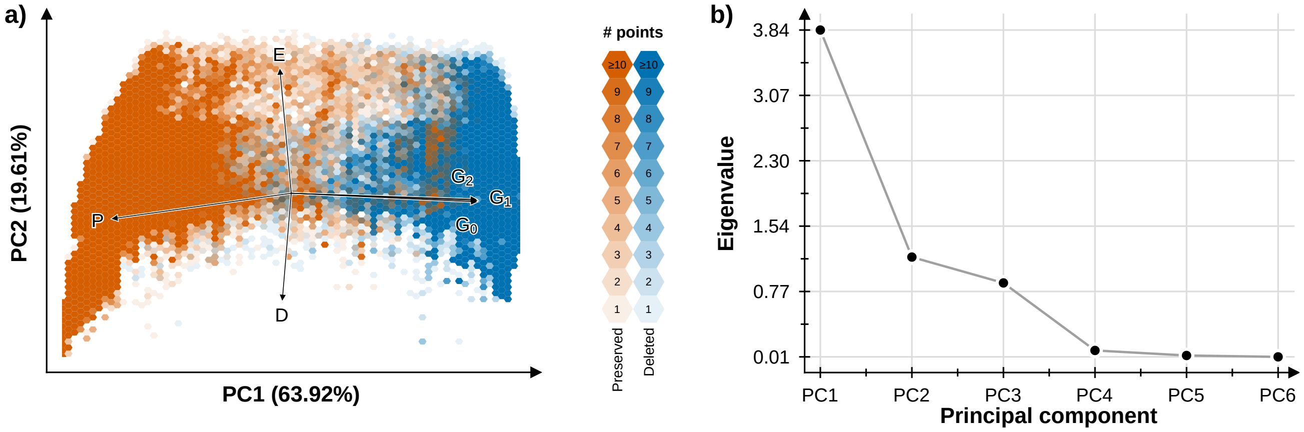

Figure 2. Results of the PCA. a) Biplot showing the density of test data columns in function of the principal component values and the component loadings. Preserved columns are shown in ⬢ orange, while deleted columns are shown in ⬢ blue. *PC*: Principal component.; G_0: Gap proportion; *P*: Percent identity; *D*: Distance from extremity; *E*: Entropy; G_1: Gap proportion (±1); G_2: Gap proportion (±2). **b)** Scree plot showing the eigenvalue (amount of explained variance) corresponding to each principal component.

An LDA (**Fig. 3**) also uses a linear transformation to project the data to a different coordinate space, but in this case each component attempts to explain as much of the difference between the two classes of data ("preserved" or "deleted") as possible.

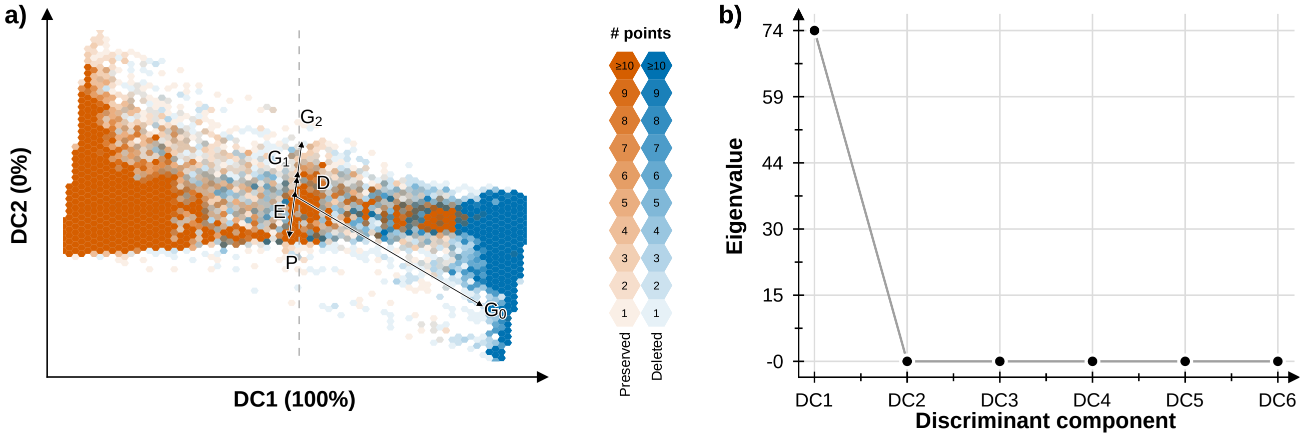

Figure 3. Results of the LDA. a) Biplot showing the density of training data columns in function of the discriminant component values and the projections of the original features in the LDA space. Preserved columns are shown in ⬢ orange, while deleted columns are shown in ⬢ blue. The - - - dashed line represents the intersection of the discriminant hyperplane with the 2D plane shown in the plot. Component abbreviations are *DC1*: Discriminant component 1; *DC2*: Discriminant component 2; the rest as in **Figure 2a**. **b)** Scree plot showing the eigenvalue (amount of explained variance) corresponding to each discriminant component.

The LDA can be used to determine whether an alignment column should be preserved or deleted, by checking whether its representation in LDA space lies closer to centroid of the preserved columns or the centroid of the deleted columns. This defines a hyperplane in the LDA coordinate space (dashed line in **Fig. 3a**), such that all columns that are located on one side of this hyperplane are preserved, and all columns that are located on the other side are deleted.

When this criterion is used to analyse the input data, 7658 columns (2.55% of the total) are incorrectly preserved or deleted (**Fig. 4**). Generally, these points should be located around the discriminant hyperplane; if many of them are located far from the discriminant plane, it might be a sign that the test dataset is internally inconsistent. Alternatively, the 6 features analysed by AliFilter may not be sufficient to capture the distinction between columns that have been preserved and those that have been deleted.

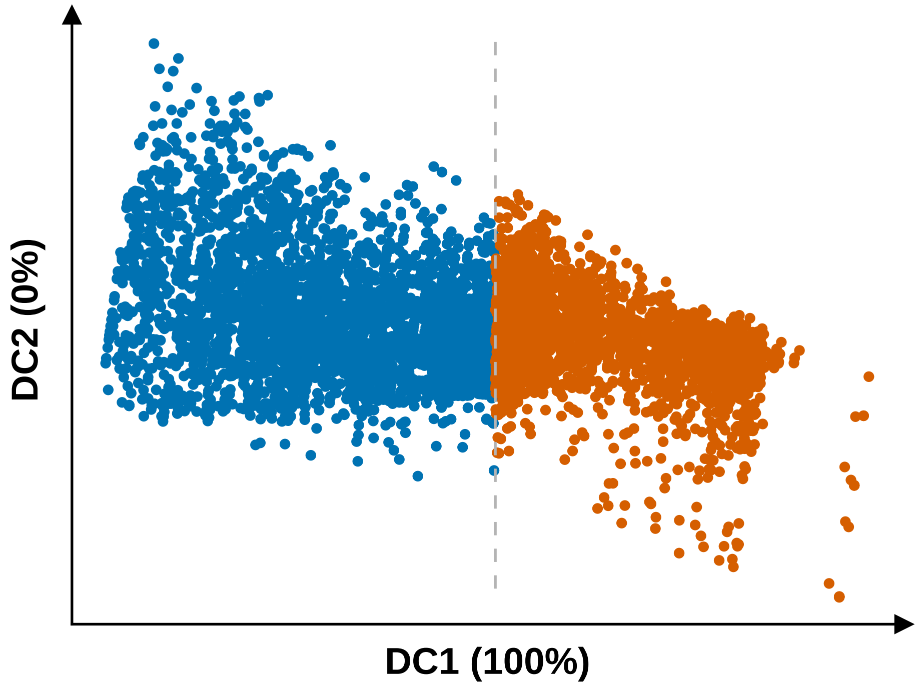

Figure 4. Incorrectly assigned input columns. This scatter plot shows the position in the LDA space of the input columns that were assigned incorrectly by the LDA analysis. Columns that were incorrectly deleted are shown in ● orange, while columns that were incorrectly preserved are shown in ● blue. The - - - dashed line represents the intersection of the discriminant hyperplane with the 2D plane shown in the plot. *DC1*: Discriminant component 1; *DC2*: Discriminant component 2.

Model analysis

The model being tested is a logistic model, which uses a linear combination of the feature values for each column to determine the log-odds that the column be preserved. The log-odds are then converted to a preservation score (ranging from 0 to 1), and columns with a preservation score lower than a specified threshold (in this case, 0.36) are deleted.

The distribution of the preservation score for each column computed using this model is shown in **Figure 5a**. When this criterion is used to analyse the test data, 6580 columns (2.19% of the total) are incorrectly preserved or deleted. The distribution of the preservation scores for these incorrectly-assigned columns is shown in **Figure 5b**.

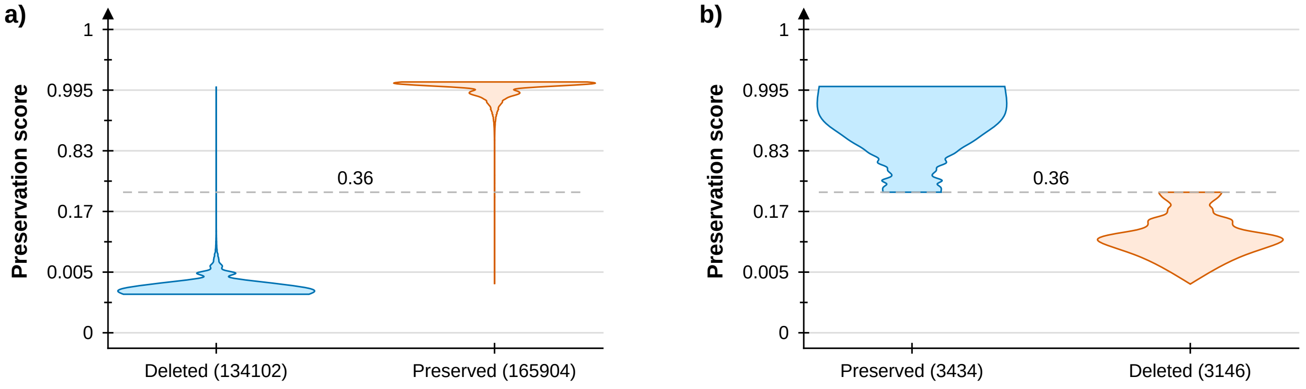

Figure 5. Logistic model results. a) Distribution of the preservation score according to the logistic model for columns that were marked as "deleted" in the input data (in ● blue, on the left) and for columns that were marked as "preserved" in the input data (in ● orange, on the right). The - - - dashed line represents the 0.36 threshold that determines whether a column is preserved or deleted according to the model. **b)** Distribution of the preservation score for columns that were incorrectly preserved (in ● blue, on the left) and for columns that were incorrectly deleted (in ● orange, on the right). The - - - dashed line represents the 0.36 threshold that determines whether a column is preserved or deleted according to the model.

The threshold value (in this case, 0.36) determines which columns are deleted or preserved. Alignment columns with a score lower than the threshold are deleted, while those with a score higher than or equal to the threshold are preserved. This determines the number of true and false positives and negatives (summarised in the confusion matrix, **Table 2**).

Performance metrics

Starting from the values in the confusion matrix, a number of metrics describing the performance of the model can be computed.

Table 2. Confusion matrix. The table shows the number of true positives (TP), false positives (FP), true negatives (TN), and false negatives (FN), when comparing the model assignments with the test data.

		Test data	
		Preserved	Deleted
Model	Preserved	TP = 162758	FP = 3434
	Deleted	FN = 3146	TN = 130668

Accuracy

The **accuracy** (A) represents the proportion of correct model assignments over the total number of assignments:

$$A = \frac{\mathrm{TP} + \mathrm{TN}}{\mathrm{TP} + \mathrm{TN} + \mathrm{FP} + \mathrm{FN}} = 0.9781$$

The accuracy score ranges from 0 to 1, with good models scoring close to 1. Note that in the case of a very imbalanced test dataset (i.e., where most columns are preserved or discarded), a trivial classifier that either preserves all columns or discards al columns might have a surprisingly high accuracy.

Matthews correlation coefficient

The **Matthews correlation coefficient** (MCC) measures the correlation of the predicted assignments with the test assignments:

$$MCC = \frac{\mathrm{TP}\cdot\mathrm{TN} - \mathrm{FP}\cdot\mathrm{FN}}{\sqrt{(\mathrm{TP}+\mathrm{FP})\cdot(\mathrm{TP}+\mathrm{FN})\cdot(\mathrm{TN}+\mathrm{FP})\cdot(\mathrm{TN}+\mathrm{FN})}} = 0.9556$$

The MCC ranges from -1 to 1, with good models scoring close to 1. Values close to 0 indicate performance close to a random classifier. A high MCC usually indicates good model performance, even when the test dataset is biased towards preserved or deleted columns; however, on a small and very biased test dataset, it is possible to obtain a low MCC even when the model performance appears relatively good (i.e., a small number of incorrectly assigned columns).

Rate metrics

The **true positive rate** (TPR, also known as *recall* or *sensitivity*) and the **positive predictive value** (PPV, also known as *precision*) are defined as:

$$TPR = \frac{\mathrm{TP}}{\mathrm{TP}+\mathrm{FN}} = 0.9810 \qquad PPV = \frac{\mathrm{TP}}{\mathrm{TP}+\mathrm{FP}} = 0.9793$$

The TPR represents the proportion of columns marked as preserved in the test set, which are also preserved by the model. The PPV represents the proportion of columns that were preserved by the model, which were actually marked as preserved within the test set.

Both of these values range from 0 to 1. A high TPR indicates that the model is able to correctly identify all the columns that should be preserved, while a high PPV indicates that all the columns preserved by the model should indeed be preserved. An ideal model should have both TPR and PPV close to 1.

The **false positive rate** (FPR) is defined as:

$$FPR = \frac{\mathrm{FP}}{\mathrm{FP}+\mathrm{TN}} = 0.0256$$

This also ranges from 0 to 1. A high FPR indicates that the model tends to preserve columns that should instead be deleted, thus an ideal model's FPR should be close to 0.

F_β score

The $\boldsymbol{F_\beta}$ **score** is a weighted harmonic mean of the TPR and PPV, defined as:

$$F_\beta = \frac{(1+\beta^2)\,\mathrm{TP}}{(1+\beta^2)\,\mathrm{TP} + \beta^2\mathrm{FN} + \mathrm{FP}} \qquad \begin{aligned} F_{0.5} &= 0.9797 \\ F_1 &= 0.9802 \\ F_2 &= 0.9807 \end{aligned}$$

The F_β score ranges from 0 to 1, with good models scoring close to 1. Note that this score does not account for the number of true negatives and therefore might be misleading in some situations.

The value of β determines the relative weight given to false positives and false negatives. Values > 1 penalise false negatives more than false positives. Values < 1 penalise false positives more than false negatives.

The F_β score curve (**Fig. 6**) has a sigmoid shape, ascending if the number of false positives is greater than the number of false negatives, and descending if the number of false negatives is greater than the number of false positives. The asymptotes of this curve are 0.9810 (for $\beta \to +\infty$) and 0.9793 (for $\beta \to 0$).

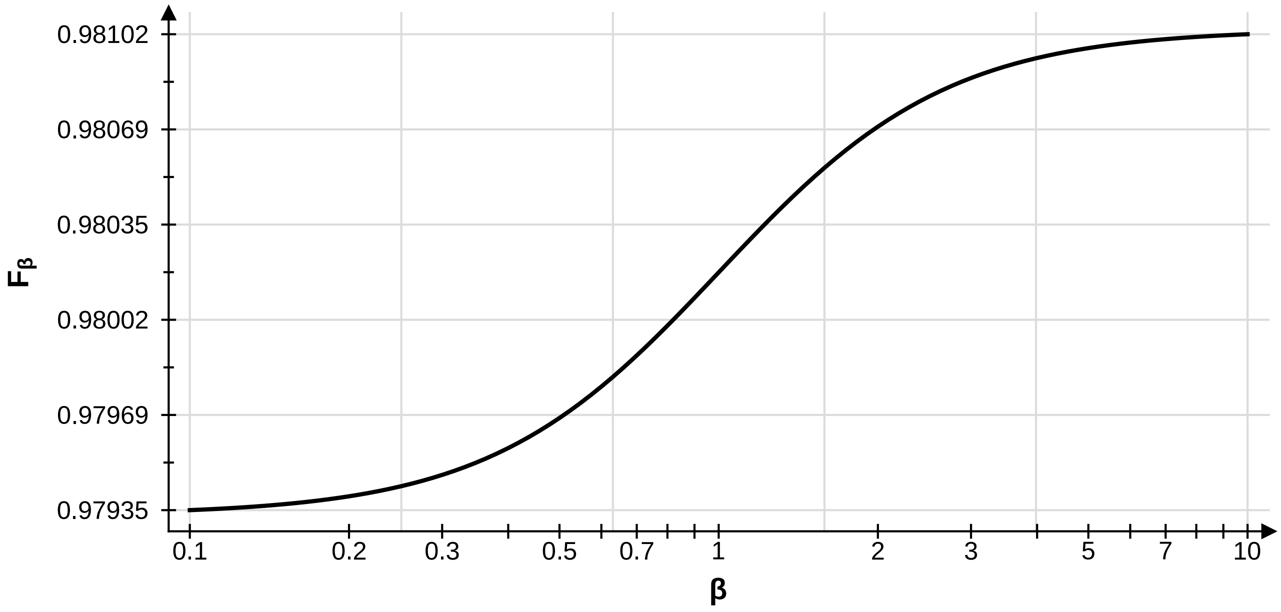

Figure 6. F_β **score curve.** The curve shows the value of F_β as a function of β.

Receiver operating characteristic

All the metrics described above are computed based on the TP, TN, FP, and FN values, and therefore they depend on the chosen threshold value. However, it might be interesting to analyse the model's performance independently of the threshold. For example, if the model performs poorly on the test dataset, a possible explanation would be that an inappropriate threshold is being used (which may indicate that the validation set is too small); however, if there is no threshold value that would improve the model's performance, this would indicate that the training set is too small (or that the features used by AliFilter are inadequate for the task).

This can be achieved by analysing the **receiver operarating characteristic** (ROC) curve (**Fig. 7**), which represents the TPR and the corresponding FPR for all threshold values. For a good model, the ROC curve should be close to the upper left corner of the plot. This is summarised by the **area under the curve** (AUC), which in this case is 0.9978 (for a good model, this should be close to 1, while a random classifier would have AUC close to 0.5).

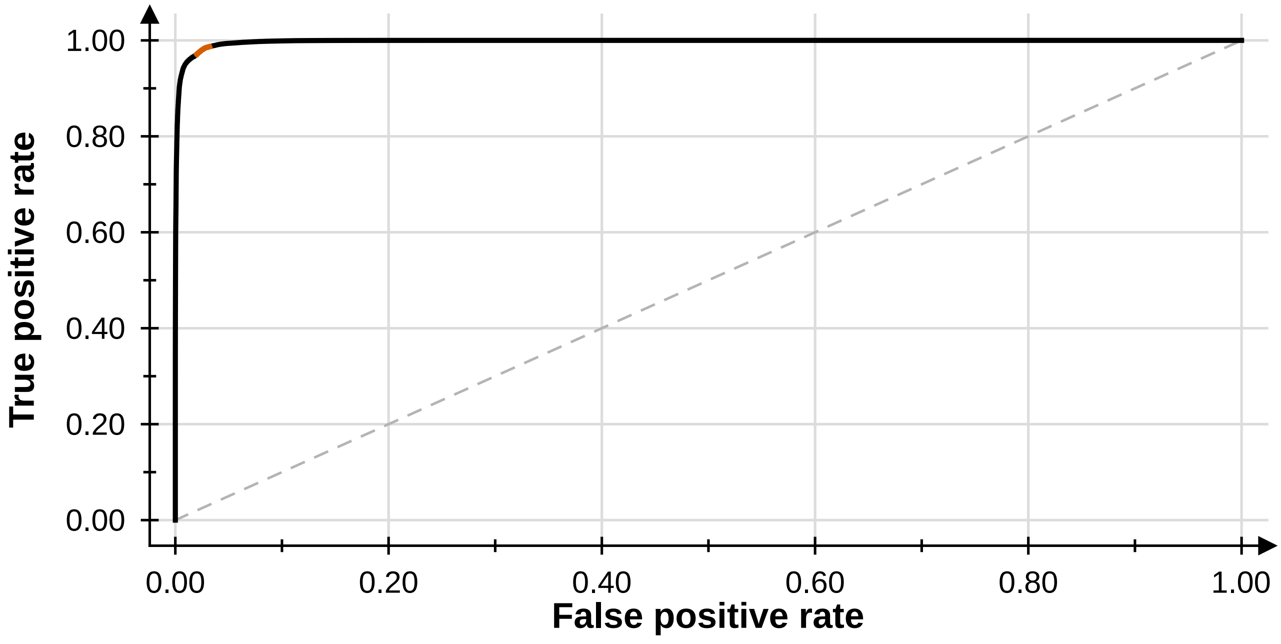

Figure 7. ROC (receiver operating characteristic) curve. The —— black line shows the ROC curve of the final model. The —— orange part of the line represents the TPR and FPR corresponding to the 0.36 threshold used for the model. The - - - dashed line represents the performance of a random classifier.

Model confidence

When classifying the alignment columns, the model assigns a score ranging between 0 and 1 to each column. For columns confidently deleted by the model, this score is close to 0; instead, for columns confidently preserved by the model, the score is close to 1. The overall confidence of the model in its assignments can be summarised using the model confidence score C, which is defined as:

$$C = 1 - \frac{4}{n} \sum_{i=1}^{n} s_i \cdot (1 - s_i)$$

Where n is the number of columns in the alignment and s_i is the confidence score for column i. For a model performing confident assignments, this score should be close to 1. In this case, it is 0.9373. Note that this score does not necessarily assess whether the model is “good” or “bad”, but just how confident it is.

Summary

The performance metric values for the final model are summarised in the table below.

**Performance metric**	**Symbol**	**Value**
Accuracy	A	0.9780671053245602
Matthews correlation coefficient	MCC	0.9556283591288784
True positive rate	TPR	0.9810372263477674
Positive predictive value	PPV	0.9793371522094927
False positive rate	FPR	0.02560737349181966
F_β score	$F_{0.5}$	0.979676695494732
F_β score	F_1	0.9801864521102331
F_β score	F_2	0.9806967394867246
Area under the ROC curve	AUC	0.9978138432331811
Model confidence	C	0.9373496548442299

Feature effects

To assess the effect of each alignment feature in determining whether a column should be deleted or preserved, the observed values for each feature were plotted against the preservation score of columns presenting that value (**Fig. 8**). To further assess the effect of individual features, additional datapoints were simulated, by considering 100 different values for each feature (ranging from the observed minimum to the observed maximum), and then randomly sampling 100 sets of values for the other features (**Fig 9**).

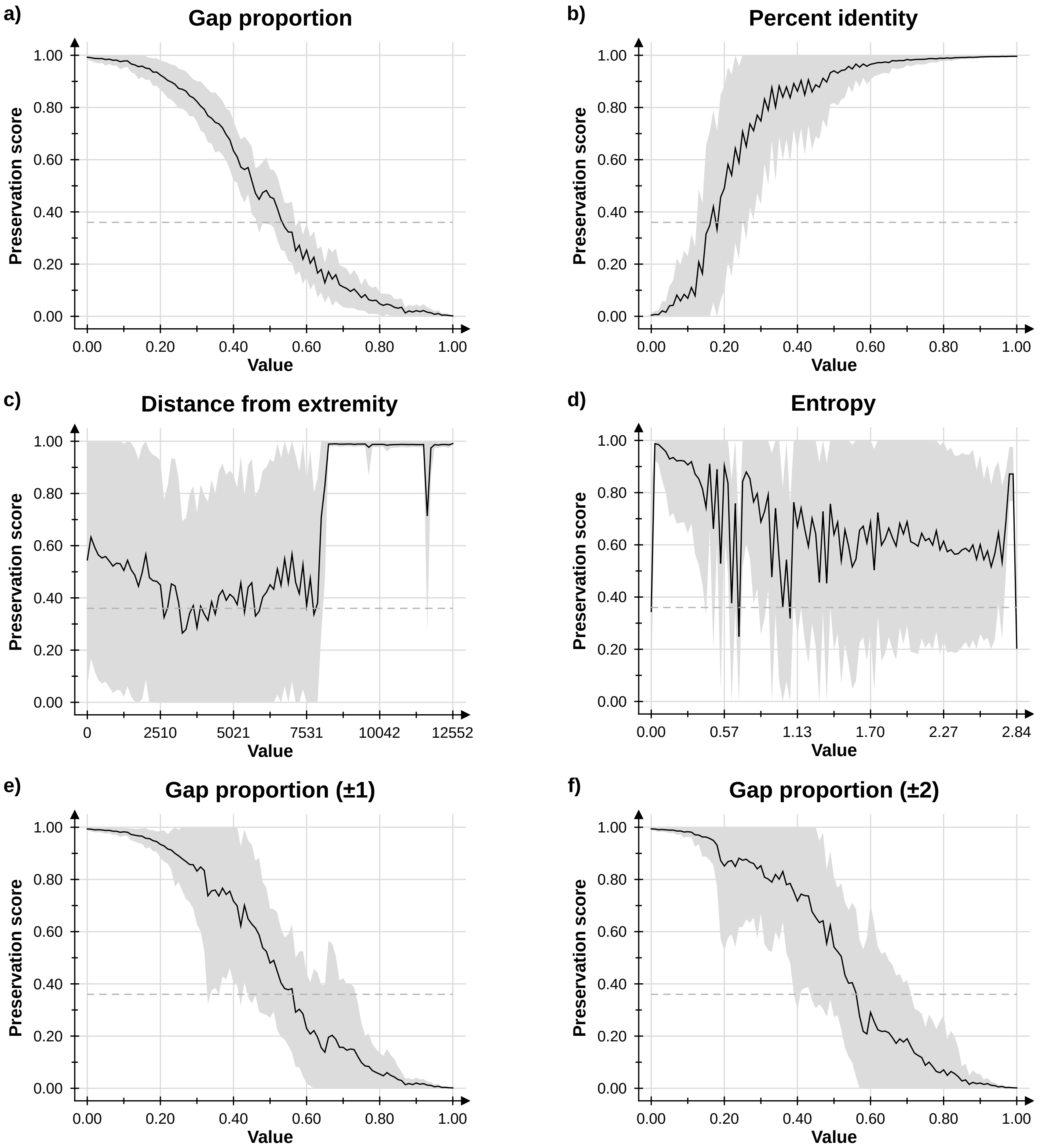

Figure 8. Effect of observed alignment feature values. Each plot shows the preservation score of the input data columns in function of the observed value for each statistic. The black lines represent the mean values, while the grey backgrounds represent the standard deviation. The - - - dashed line represents the 0.36 threshold that determines whether a column is preserved or deleted according to the model.

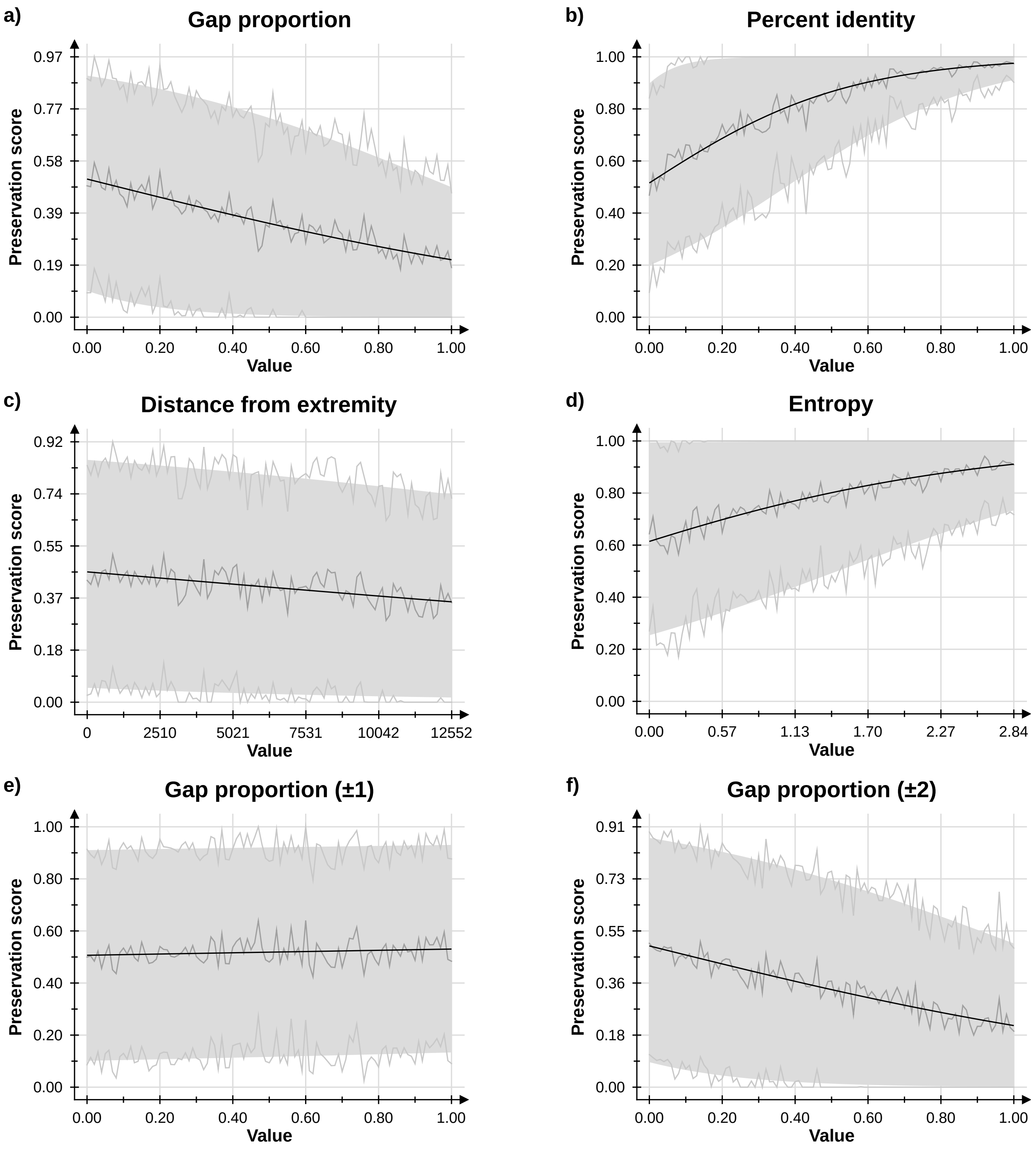

Figure 9. Effect of simulated alignment feature values. Each plot shows the preservation score of a simulated data column as a function of the sampled values for each statistic. In each plot, the black line represents the mean values, while the grey background represents the standard deviation. To produce these plots, the value for the “focal” feature was fixed along a the range of observed values. Then, random values were sampled for each feature, according to its observed distribution. The logistic model was then used to determine the preservation score for each combination of random feature values. Finally, logistic curves were used to interpolate the relationship between the preservation score and the feature values. The grey lines show the computed values, while the black line and the grey background show the interpolated values.
