# Supplementary material for: AliFilter: a machine learning approach to alignment filtering
Source: Mol Biol Evol. 2026 Apr 10;43(4):msag097. doi: 10.1093/molbev/msag097 (PMC13108598; doi:10.1093/molbev/msag097)
Supplement: msag097_Supplementary_Data [file msag097_supplementary_data.zip › alifilter.validation_report.pdf]

Model validation report

Created by AliFilter version 1.0.0 on 24th Nov, 2024 at 18:58.

MD5 checksum of the model this report refers to: `E95D52E3F38227FDA87BC60EBBE59E34` (feature signature: `8D2F81A3625201DA548E08978D516E0C`)

This report may be included in any analysis using this model.

This report is machine-readable. If the report file is called `report.pdf`, you can export the validated model to a file called `model.json` by running:

- On a Unix machine:

```
grep -a "@model" report.pdf | sed "s/@model//g" > model.json
```

- On a Windows machine (within a PowerShell environment):

```
findstr "@model" report.pdf | %{$_ -replace "@model",""} > model.json
```

Validation data analysis

The model was validated using data from 230674 alignment columns, of which 117716 (51.03%) were preserved and 112958 (48.97%) were deleted (**Fig. 1**). For each alignment column, 6 features were computed (**Table 1**), which were analysed in a Principal Component Analysis (PCA) and in a Linear Discriminant Analysis (LDA).

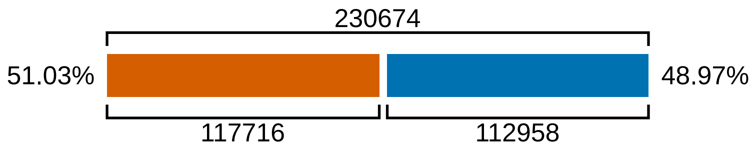

Figure 1. Proportion of preserved columns. The figure shows the proportion of data columns that were preserved (in ■ orange, on the left) or deleted (in ■ blue, on the right).

Table 1. Alignment features. The table lists the features that have been computed for each alignment column, including a brief description and the observed range, mean and standard deviation (SD) for each of them.

Name	Description	Values
Gap proportion	Proportion of sequences that have a gap in the column.	*Range*: 0 - 0.9997<br>*Mean*: 0.4979<br>*SD*: 0.4531
Percent identity	Frequency of the most common residue in the alignment column, excluding gaps.	*Range*: 0.0003 - 1<br>*Mean*: 0.4255<br>*SD*: 0.4115
Distance from extremity	Number of residues between the column and the closest extremity (start or end) of the alignment.	*Range*: 0 - 13786<br>*Mean*: 2763.5489<br>*SD*: 3312.3417
Entropy	Shannon entropy for the residue frequencies in the column, excluding gaps.	*Range*: 0 - 2.8319<br>*Mean*: 0.3656<br>*SD*: 0.4992
Gap proportion (±1)	Average of the proportion of gaps between the column, 1 preceding column(s), and 1 subsequent columns.	*Range*: 0 - 0.9997<br>*Mean*: 0.4979<br>*SD*: 0.436
Gap proportion (±2)	Average of the proportion of gaps between the column, 2 preceding column(s), and 2 subsequent columns.	*Range*: 0 - 0.9997<br>*Mean*: 0.4979<br>*SD*: 0.4288

A PCA (**Fig. 2**) uses a linear transformation to transform the data to a coordinate system where each coordinate (component) explains as much of the variance of the data as possible, while being orthogonal to the previous components. This is useful to show the distribution of the input data, but a PCA, on its own, cannot be used to decide whether an alignment column should be preserved or not.

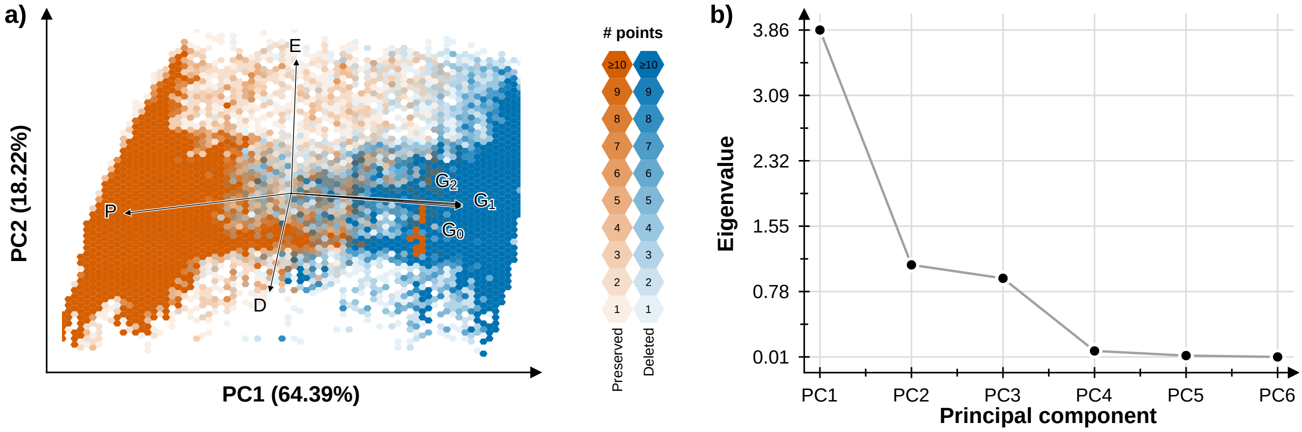

Figure 2. Results of the PCA. a) Biplot showing the density of test data columns in function of the principal component values and the component loadings. Preserved columns are shown in ⬢ orange, while deleted columns are shown in ⬢ blue. *PC*: Principal component.; G_0: Gap proportion; *P*: Percent identity; *D*: Distance from extremity; *E*: Entropy; G_1: Gap proportion (±1); G_2: Gap proportion (±2). **b)** Scree plot showing the eigenvalue (amount of explained variance) corresponding to each principal component.

An LDA (**Fig. 3**) also uses a linear transformation to project the data to a different coordinate space, but in this case each component attempts to explain as much of the difference between the two classes of data ("preserved" or "deleted") as possible.

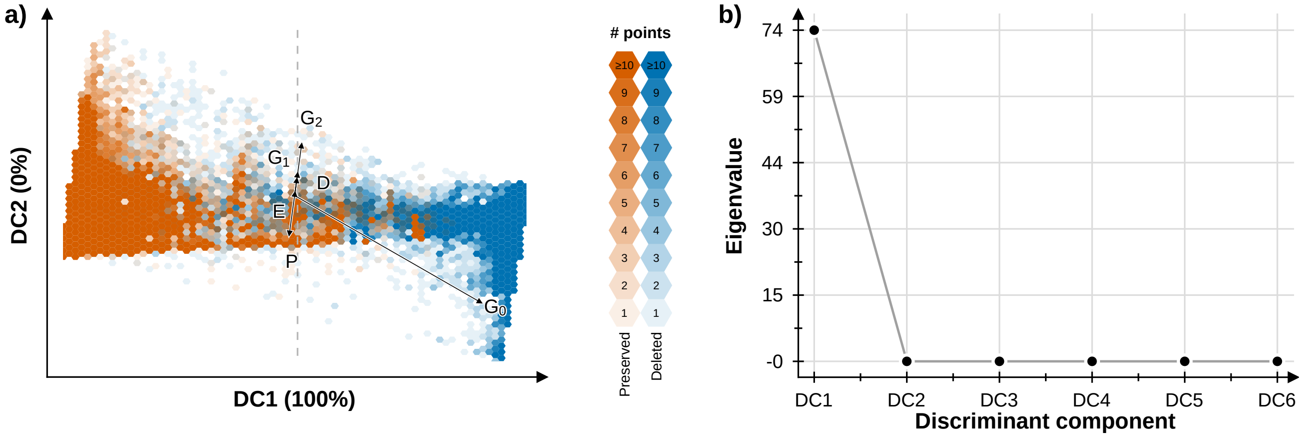

Figure 3. Results of the LDA. a) Biplot showing the density of training data columns in function of the discriminant component values and the projections of the original features in the LDA space. Preserved columns are shown in ⬢ orange, while deleted columns are shown in ⬢ blue. The - - - dashed line represents the intersection of the discriminant hyperplane with the 2D plane shown in the plot. Component abbreviations are *DC1*: Discriminant component 1; *DC2*: Discriminant component 2; the rest as in **Figure 2a**. **b)** Scree plot showing the eigenvalue (amount of explained variance) corresponding to each discriminant component.

The LDA can be used to determine whether an alignment column should be preserved or deleted, by checking whether its representation in LDA space lies closer to centroid of the preserved columns or the centroid of the deleted columns. This defines a hyperplane in the LDA coordinate space (dashed line in **Fig. 3a**), such that all columns that are located on one side of this hyperplane are preserved, and all columns that are located on the other side are deleted.

When this criterion is used to analyse the input data, 7061 columns (3.06% of the total) are incorrectly preserved or deleted (**Fig. 4**). Generally, these points should be located around the discriminant hyperplane; if many of them are located far from the discriminant plane, it might be a sign that the validation dataset is internally inconsistent. Alternatively, the 6 features analysed by AliFilter may not be sufficient to capture the distinction between columns that have been preserved and those that have been deleted.

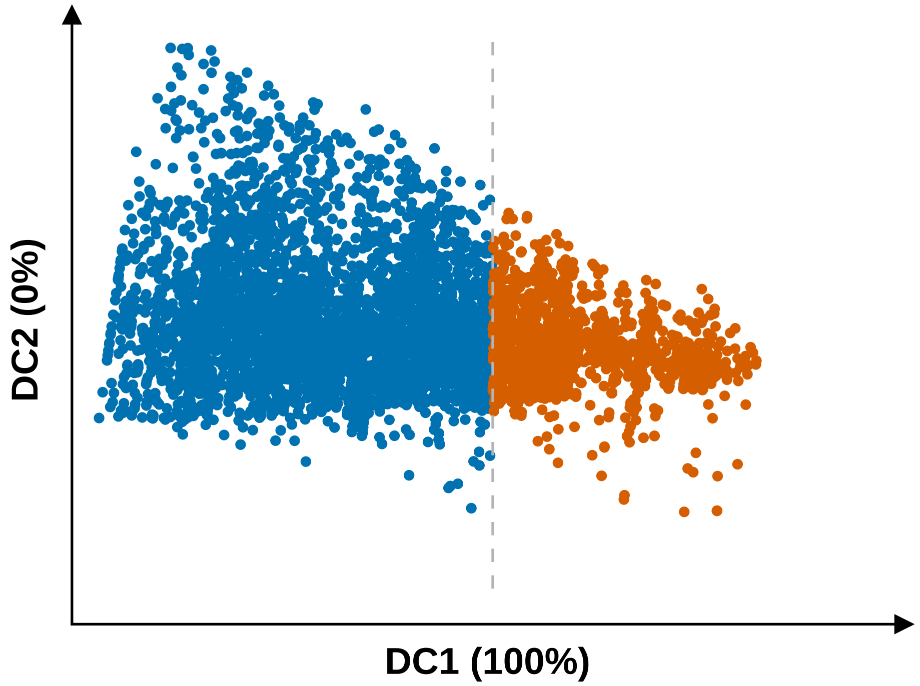

Figure 4. Incorrectly assigned input columns. This scatter plot shows the position in the LDA space of the input columns that were assigned incorrectly by the LDA analysis. Columns that were incorrectly deleted are shown in ● orange, while columns that were incorrectly preserved are shown in ● blue. The - - - dashed line represents the intersection of the discriminant hyperplane with the 2D plane shown in the plot. *DC1*: Discriminant component 1; *DC2*: Discriminant component 2.

Model validation

The model being validated is a logistic model, which uses a linear combination of the feature values for each column to determine the log-odds that the column be preserved. The log-odds are then converted to a preservation score (ranging from 0 to 1), and columns with a preservation score lower than a specified threshold (e.g., 0.5) are deleted.

The confidence of the model in the classification of each alignment column can be assessed by performing row-wise bootstrap replicates (note that this is different from most phylogenetic analyses, where bootstrap is performed column-wise). Each replicate column can be assessed by the logistic model, and a final assessment can be performed by counting how many replicates pass the preservation threshold and comparing this with a bootstrap replicate threshold.

Three parameters were thus tuned by cross-validation:

- The logistic model threshold (range: 0 - 1).
- The number of bootstrap replicates (range: 0 - 1000).
- The bootstrap threshold (range: 0 - 1).

For each combination of parameters, the Matthews correlation coefficient MCC was computed. This is defined as:

$$MCC = \frac{\mathrm{TP} \cdot \mathrm{TN} - \mathrm{FP} \cdot \mathrm{FN}}{\sqrt{(\mathrm{TP} + \mathrm{FP}) \cdot (\mathrm{TP} + \mathrm{FN}) \cdot (\mathrm{TN} + \mathrm{FP}) \cdot (\mathrm{TN} + \mathrm{FN})}}$$

Where TP is the number of true positives, TN is the number of true negatives, FP is the number of false positives, and FN is the number of false negatives.

The MCC ranges from -1 to 1, with good models scoring close to 1. Values close to 0 indicate performance similar to a random classifier.

An additional score S was also computed, which takes into account the fact that while using bootstrap replicates can improve the prediction score, computing them requires time. This is defined as:

$$S = MCC - \frac{b}{100} \cdot 0.005$$

Where b is the number of bootstrap replicates. This ensures that an additional 100 bootstrap replicates are only performed if they improve the prediction score by at least 0.005.

The following plots show the value of the prediction score as a function of the two threshold parameters, for each number of bootstrap replicates. **Table 2** summarises the best scores and the corresponding threshold values for each number of bootstrap replicates.

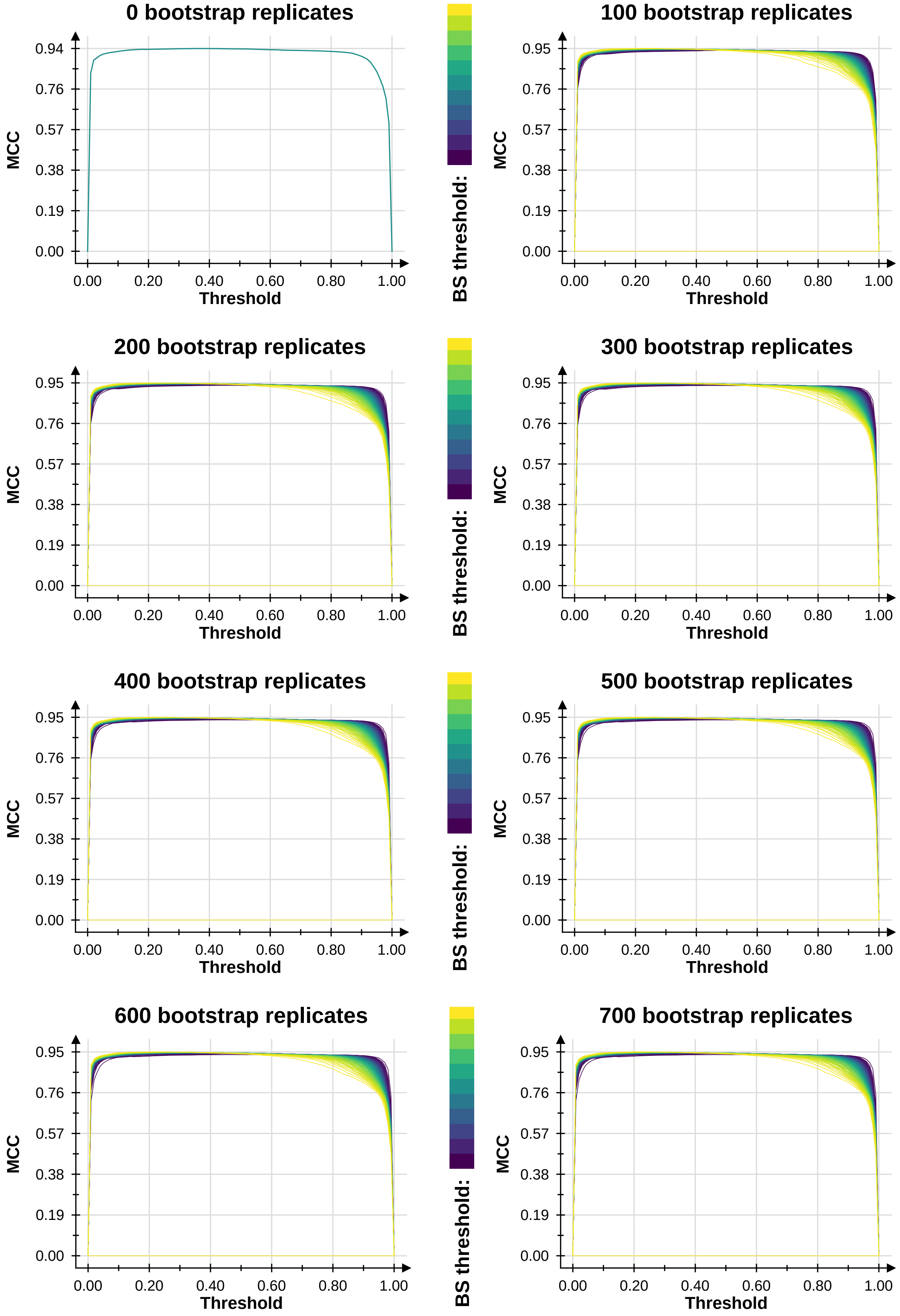
0 bootstrap replicates
100 bootstrap replicates
200 bootstrap replicates
300 bootstrap replicates
400 bootstrap replicates
500 bootstrap replicates
600 bootstrap replicates
700 bootstrap replicates
MCC
0.00
0.19
0.38
0.57
0.76
0.94
0.95
Threshold
0.00
0.20
0.40
0.60
0.80
1.00
BS threshold:

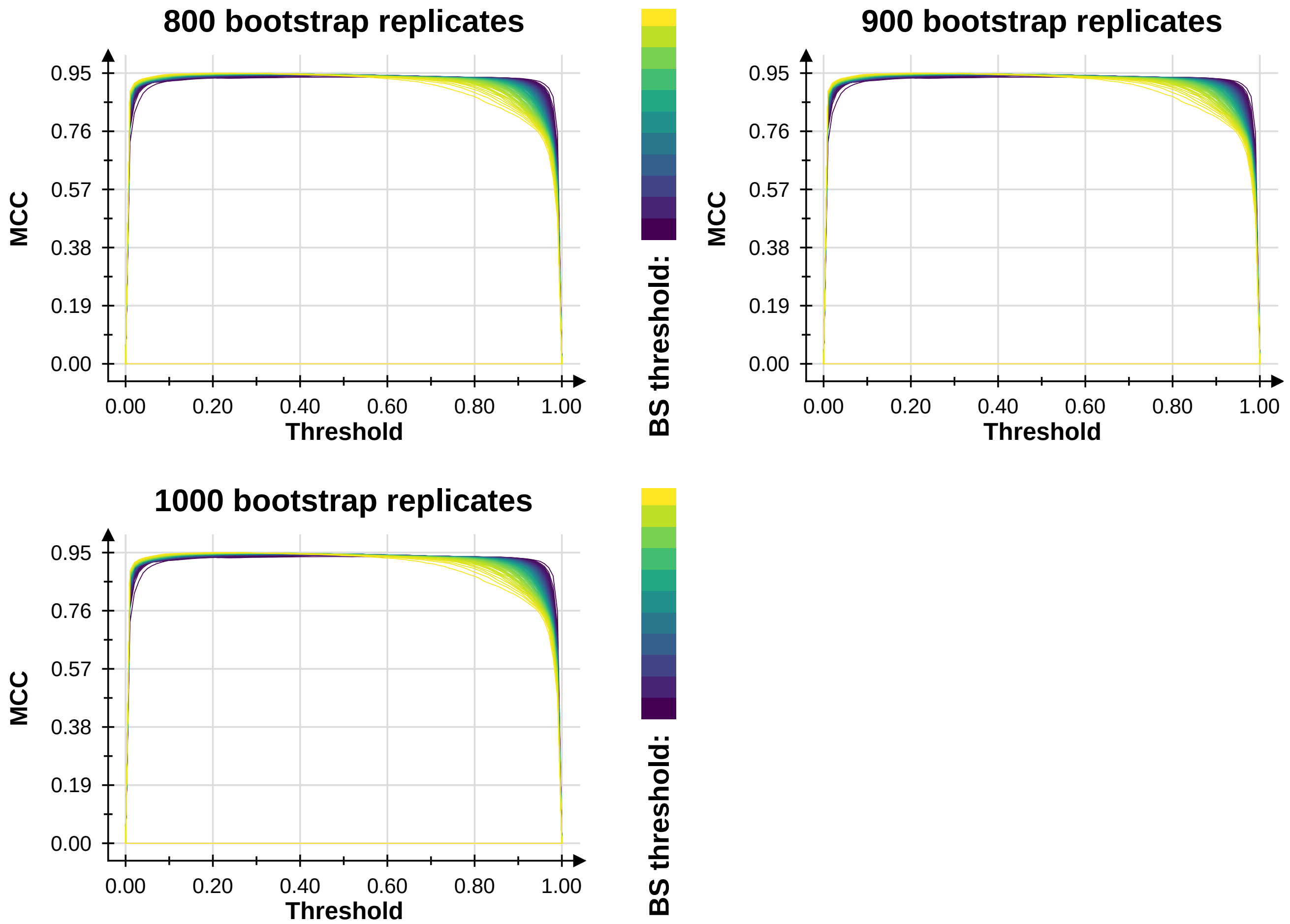

Table 2. Scores and threshold values. The table shows for each number of bootstrap replicates, the maximum value for the MCC score and the corresponding S score, logistic model threshold, and bootstrap threshold.

BS replicates	Score	MCC	Threshold	BS threshold
0	0.943940	0.943940	0.36	0.50
100	0.941737	0.946737	0.21	0.98
200	0.936885	0.946885	0.21	0.99
300	0.931868	0.946868	0.21	0.99
400	0.926692	0.946692	0.22	0.98
500	0.921822	0.946822	0.20	0.99
600	0.916938	0.946938	0.21	0.99
700	0.911851	0.946851	0.21	0.99
800	0.906826	0.946826	0.21	0.99
900	0.901824	0.946824	0.22	0.98
1000	0.896920	0.946920	0.21	0.98

The overall best MCC score (0.9469383252684299, corresponding S score 0.9169383252684299) was obtained for 600 bootstrap replicates, with logistic model threshold 0.21 and bootstrap threshold 0.99.

The overall best S score (0.9439399391100289, corresponding MCC score 0.9439399391100289) was obtained for 0 bootstrap replicates, with logistic model threshold 0.36 and bootstrap threshold 0.5.

These parameter values have been stored with the validated model.
