# Supplementary material for: AliFilter: a machine learning approach to alignment filtering
Source: Mol Biol Evol. 2026 Apr 10;43(4):msag097. doi: 10.1093/molbev/msag097 (PMC13108598; doi:10.1093/molbev/msag097)
Supplement: msag097_Supplementary_Data [file msag097_supplementary_data.zip › AliFilter_SI_FINAL_Clean_25Feb2026.pdf]

Supplementary Information

AliFilter: a Machine Learning Approach to Alignment Filtering

Giorgio Bianchini[1,2*], Rui Zhu[1], Francesco Cicconardi[3], Edmund RR Moody[4,5]

[1] School of Geographical Sciences, University of Bristol, United Kingdom

[2] Department of Life Sciences, University of Bath, United Kingdom

[3] School of Biological Sciences, University of Bristol, United Kingdom

[4] School of Chemistry, University of Bristol, United Kingdom

[5] Departament de Genètica, Microbiologia i Estadística, Universitat de Barcelona, Spain

[*] Address correspondence to:

Giorgio Bianchini

Department of Life Sciences, University of Bath

BA2 7AX Claverton Down, United Kingdom

gb2177@bath.ac.uk

Supplementary Methods

Datasets

Model training, validation and testing

Eight primary datasets were used to train AliFilter models and assess the performance of the program (**Table 2**). In addition, five further datasets were included:

three secondary datasets obtained by merging the primary datasets, and two independent datasets used to test the AliFilter model against other types of data that were not used in the development of the software.

Dataset 1 (“Cyanobacteria (phylogenomic)”) consists of 139 alignments of manually curated cyanobacterial genes from a previously published study (Bianchini et al. 2024). Each alignment contains approximately 200 amino acid sequences from Cyanobacteria and some outgroup genomes (Vampirovibrionia, Serycytochromatia, and other Terrabacteria).

Dataset 2 (“Cyanobacteria (BUSCO)”) consists of 40 alignments randomly selected out of a set of 773 cyanobacterial single copy orthologs retrieved by BUSCO v5.4.3 (Manni et al. 2021) with the cyanobacteria_odb10 dataset. Each alignment contains approximately 3000 amino acid sequences from genomes of Cyanobacteria.

Dataset 3 (“Rhodobacteraceae (BUSCO)”) also consists of 40 alignments, randomly selected out of 833 single copy orthologs retrieved by BUSCO v5.4.3 with the rhodobacterales_odb10 dataset. Each alignment contains approximately 150 amino acid sequences from genomes of Rhodobacteraceae.

Dataset 4 (“Prokaryotes (COG)”) consists of 54 alignments of the vertically evolving single-copy marker genes identified in (Moody et al. 2022), sampled from 350 archaea and 350 bacteria representing the breadth of prokaryotic diversity.

Dataset 5 (“Collembola (BUSCO)”) consists of 40 alignments, selected using the --suggest function of AliFilter out of 1,013 single copy orthologs retrieved by BUSCO v5.4.3 with the arthropoda_odb10 dataset. Each alignment was produced using a similar pipeline as in (Cicconardi et al. 2020; Cicconardi et al. 2023). More specifically, the nucleotide sequences for each ortholog were processed before the alignment with PREQUAL v1.02 [-

pptype all] (Whelan et al. 2018), then codon-wise aligned with MACSE v2.03 (Ranwez et al. 2018), and subsequently re-filtered with HmmCleaner (Di Franco et al. 2019); sequences were then de-aligned and the final alignment was performed using MAFFT (Katoh and Standley 2013) as described in the main text. Each alignment has a variable set of taxa from a total of 149 species. The dataset was built using genomic data available on the NCBI (O'Leary et al. 2016) and MetaInvert databases (Collins et al. 2023), as well as additional data under manuscript preparation.

Dataset 6 ("Formicidae (BUSCO)") consists of 40 alignments, selected using the --suggest function of AliFilter out of 5,991 single copy orthologs retrieved by BUSCO v5.4.3 with the hymenoptera_odb10 dataset. Each alignment was produced using the same pipeline as Dataset 5 and contains a variable set of taxa from a total of 71 species. The dataset was built using genomic data available on NCBI (O'Leary et al. 2016), as well as additional data under manuscript preparation.

Dataset 7 ("Heliconiini (scOG)") consists of 40 alignments, selected using the --suggest function of AliFilter out of 3,393 single copy orthologs identified in (Cicconardi et al. 2023). Alignments contain a variable set of taxa from a total of 63 species and were built as in Datasets 5 and 6.

Dataset 8 ("rRNA (SILVA)") consists of 58 alignments of small subunit (SSU) and large subunit (LSU) rRNA sequences, retrieved from the SILVA database (Quast et al. 2013). Each alignment contains between 42 and 2000 rRNA sequences from a single group (phylum, class, or order) of prokaryotes or eukaryotes.

Dataset 12 ("Animals (Mitochondria)") consists of 35 alignments of proteins (n = 13), tRNA (n = 20), and rRNA (n = 2) encoded in the mitochondrial genome of 199 metazoan species (Boore 1999; Lavrov and Pett 2016). This dataset was assembled by first searching for 'genome' in the GenBank Nucleotide database (Sayers et al. 2025) and

filtering for sequences from ‘Animals’ and ‘Mitochondrion’; the ‘accession2taxid’ resource from the NCBI taxonomy database (Schoch et al. 2020) was then used to assign a taxonomy ID to the resulting 247 272 hits, which were clustered by class. The sequences from up to three species per class were selected, after excluding sequences whose length deviated substantially from the median of each class. Annotations in GenBank format were downloaded for the 199 selected sequences, and the ‘product’ field was used to identify protein-coding gene features as well as tRNAs and rRNAs (with manual revision to address conflicting nomenclature in the annotation – e.g., ‘cytochrome c oxidase subunit 2’, ‘cytochrome c oxidase subunit II’, and ‘cytochrome oxitase *[sic]* subunit II’). Note that most animal mitochondrial genomes encode two isoaccepting tRNAs for leucine and serine that cannot be easily distinguished (Rawlings et al. 2003); in our dataset these are included in a single alignment. Since we are not performing downstream phylogenetic analyses, this is not problematic for our purposes.

Dataset 13 (“*Caudoviricetes* (VOG)”) consists of 73 alignments of viral protein sequences from viruses of the class *Caudoviricetes*. These were assembled from the Virus Orthologous Groups (VOG) Database, release 232 (Trgovec-Greif et al. 2024), by retrieving the full set of VOGs and preserving only sequences belonging to *Caudoviricetes*. VOGs with fewer than 500 *Caudoviricetes* sequences were removed from the analysis, and the remaining 73 VOGs were re-aligned using MAFFT with the --localpair –maxiterate 1000 options (Katoh and Standley 2013).

Large-scale benchmarks

To benchmark AliFilter’s performance, we assembled a large-scale dataset, consisting of the SSU and LSU Ref NR99 alignments (across all domains of cellular life) from release 138.2 of the SILVA database (Quast et al. 2013), as well as alignments of the (bacterial) bac120 markers from release 226 of the Genome Taxonomy Database (GTDB)

(Parks et al. 2022). The SILVA alignments were downloaded from the database, and dots ('.') gap symbols were replaced with dashes ('-'); the alignments were otherwise untouched, and the sequences were not realigned. For the GTDB bac120 dataset, instead, the GTDB only provides unaligned sequences and partial sequences aligned by HMMer (Eddy 2011), which exclude regions (e.g., indels) that do not align well with the query HMM. To obtain full-length alignments, we thus downloaded the unaligned sequences and aligned them using MAFFT with the --auto option (since the sequence files were too large to process with --localpair --maxiterate 1000).

Simulated dataset

To examine the effect of alignment filtering on alignments with a known underlying phylogeny, we generated a simulated dataset. To produce a 'realistic' dataset, we downloaded the bacterial reference tree from release 226 of the GTDB database (Parks et al. 2022) and extracted the subtree for the phylum Gemmatimonadota (comprising 1398 representative genomes); we then collected the sequences for the 120 marker genes used by the GTDB and aligned them using MAFFT (Katoh and Standley 2013) with the --localpair --maxiterate 1000 options (three markers were deleted, as they were present in fewer than 100 genomes). We also produced a subtree for each marker, obtained by taking the full Gemmatimonadota tree and removing taxa for which the marker was not available. IQ-TREE's --alisim option (Minh et al. 2020; Ly-Trong et al. 2022) was used to 'mimic' (-s option) each of the 117 alignments, under an LG+R8 model. This process allowed us to produce a set of realistic alignments to use as a benchmark reference. We then removed all gaps from the simulated sequences, re-aligned them using MAFFT with the --localpair --maxiterate 1000 options, and filtered the resulting alignments using the various tools. Finally, we used IQ-TREE to perform partitioned maximum-likelihood analyses for each set of alignments (simulated, re-aligned, and filtered with each tool), in

which each gene was assigned to its own partition evolving under an LG+R8 model, with all partitions sharing the same branch lengths but with a different evolution rate (-p option of IQ-TREE). Three replicates were run for each analysis, with 1000 ultrafast bootstrap replicates (Hoang et al. 2018), and the resulting trees were compared.

Phylogenetic tree distance metrics

To compare phylogenetic trees obtained using alignments filtered with different methods, we employed two distance metrics: the Robinson-Foulds (RF) distance (Robinson and Foulds 1981), and the Frobenius distance (FD) computed on normalised patristic distance matrices. To compute the FD between two trees containing n taxa (**Fig. S12a**), each tree needs to be converted into an $n \times n$ patristic distance matrix M (i.e., a symmetric matrix where each entry $M_{i,j}$ is the sum of the branch lengths on the shortest path between i and j). The distance between the two trees is then computed as the Frobenius norm of the difference between the two patristic distance matrices. Additionally, as we noted that different filtering methods produce different estimates for the tree length (**Table S2**), all trees were rescaled so that the total length (sum of all branch lengths) of each tree was equal to 1 (**Fig. S12a**). This allowed us to compare the overall “shape” of the trees, rather than the raw branch length values.

The RF distance only assesses tree topology and is thus informative if branch lengths are not going to affect further analyses or the interpretation of the tree (e.g., if the tree only serves as a topological constraint for a molecular clock analysis). On the other hand, the FD considers all pairwise patristic distances between taxa on the tree, and thus assesses congruence in both branch lengths and (implicitly) topology. As a result, these two metrics can produce different representations of the tree space (**Fig. S12b**).

References

Bianchini G, Hagemann M, Sánchez-Baracaldo P. 2024. Stochastic Character Mapping, Bayesian Model Selection, and Biosynthetic Pathways Shed New Light on the Evolution of Habitat Preference in Cyanobacteria. *Syst. Biol.* [Internet]:syae025. Available from: https://doi.org/10.1093/sysbio/syae025

Boore JL. 1999. Animal mitochondrial genomes. *Nucleic Acids Res.* [Internet] 27:1767–1780. Available from: https://dx.doi.org/10.1093/nar/27.8.1767

Cicconardi F, Krapf P, D'Annessa I, Gamisch A, Wagner HC, Nguyen AD, Economo EP, Mikheyev AS, Guenard B, Grabherr R, et al. 2020. Genomic Signature of Shifts in Selection in a Subalpine Ant and Its Physiological Adaptations. *Mol. Biol. Evol.* [Internet] 37:2211–2227. Available from: https://dx.doi.org/10.1093/molbev/msaa076

Cicconardi F, Milanetti E, Pinheiro de Castro EC, Mazo-Vargas A, Van Belleghem SM, Ruggieri AA, Rastas P, Hanly J, Evans E, Jiggins CD, et al. 2023. Evolutionary dynamics of genome size and content during the adaptive radiation of Heliconiini butterflies. *Nature Communications 2023 14:1* [Internet] 14:1–24. Available from: https://www.nature.com/articles/s41467-023-41412-5

Collins G, Schneider C, Boštjančić LL, Burkhardt U, Christian A, Decker P, Ebersberger I, Hohberg K, Lecompte O, Merges D, et al. 2023. The MetaInvert soil invertebrate genome resource provides insights into below-ground biodiversity and evolution. *Communications Biology 2023 6:1* [Internet] 6:1–12. Available from: https://www.nature.com/articles/s42003-023-05621-4

Eddy SR. 2011. Accelerated Profile HMM Searches. *PLoS Comput. Biol.* [Internet] 7:e1002195. Available from: https://journals.plos.org/ploscompbiol/article?id=10.1371/journal.pcbi.1002195

Di Franco A, Poujol R, Baurain D, Philippe H. 2019. Evaluating the usefulness of alignment filtering methods to reduce the impact of errors on evolutionary inferences. *BMC Evol. Biol.* [Internet] 19:1–17. Available from: https://link.springer.com/articles/10.1186/s12862-019-1350-2

Herlihy M, Shavit N, Luchangco V, Spear M. 2021. Chapter 1 - Introduction. In: Herlihy M, Shavit N, Luchangco V, Spear M, editors. The Art of Multiprocessor Programming (Second Edition). Boston: Morgan Kaufmann. p. 1–18. Available from: https://www.sciencedirect.com/science/article/pii/B9780124159501000094

Higinbotham P. 2019. PowerShell ForEach-Object Parallel Feature. Available from: https://devblogs.microsoft.com/powershell/powershell-foreach-object-parallel-feature/

Hoang DT, Chernomor O, von Haeseler A, Minh BQ, Vinh LS. 2018. UFBoot2: Improving the Ultrafast Bootstrap Approximation. *Mol. Biol. Evol.* [Internet] 35:518–522. Available from: https://academic.oup.com/mbe/article/35/2/518/4565479

Kapli P, Natsidis P, Leite DJ, Fursman M, Jeffrie N, Rahman IA, Philippe H, Copley RR, Telford MJ. 2025. Lack of support for Deuterostomia prompts reinterpretation of the first Bilateria. *Sci. Adv.* [Internet] 7:eabe2741. Available from: https://doi.org/10.1126/sciadv.abe2741

Katoh K, Standley DM. 2013. MAFFT Multiple Sequence Alignment Software Version 7: Improvements in Performance and Usability. *Mol. Biol. Evol.* [Internet] 30:772–780. Available from: https://academic.oup.com/mbe/article-lookup/doi/10.1093/molbev/mst010

Lavrov D V., Pett W. 2016. Animal Mitochondrial DNA as We Do Not Know It: mt-Genome Organization and Evolution in Nonbilaterian Lineages. *Genome Biol. Evol.* [Internet] 8:2896–2913. Available from: https://dx.doi.org/10.1093/gbe/evw195

Ly-Trong N, Naser-Khdour S, Lanfear R, Minh BQ. 2022. AliSim: A Fast and Versatile Phylogenetic Sequence Simulator for the Genomic Era. *Mol. Biol. Evol.* [Internet] 39. Available from: https://dx.doi.org/10.1093/molbev/msac092

Manni M, Berkeley MR, Seppey M, Simão FA, Zdobnov EM. 2021. BUSCO Update: Novel and Streamlined Workflows along with Broader and Deeper Phylogenetic Coverage for Scoring of Eukaryotic, Prokaryotic, and Viral Genomes. *Mol. Biol. Evol.* [Internet] 38:4647–4654. Available from: https://dx.doi.org/10.1093/molbev/msab199

Minh BQ, Schmidt HA, Chernomor O, Schrempf D, Woodhams MD, Von Haeseler A, Lanfear R, Teeling E. 2020. IQ-TREE 2: New Models and Efficient Methods for Phylogenetic Inference in the Genomic Era. *Mol. Biol. Evol.* [Internet] 37:1530–1534. Available from: https://dx.doi.org/10.1093/molbev/msaa015

Moody ERR, Mahendrarajah TA, Dombrowski N, Clark JW, Petitjean C, Offre P, Szöllősi GJ, Spang A, Williams TA. 2022. An estimate of the deepest branches of the tree of life from ancient vertically evolving genes. *Elife* 11.

O'Leary NA, Wright MW, Brister JR, Ciufo S, Haddad D, McVeigh R, Rajput B, Robbertse B, Smith-White B, Ako-Adjei D, et al. 2016. Reference sequence (RefSeq) database at NCBI: current status, taxonomic expansion, and functional annotation. *Nucleic Acids Res.* [Internet] 44:D733–D745. Available from: http://www.ncbi.nlm.nih.gov/pubmed/26553804

Parks DH, Chuvochina M, Rinke C, Mussig AJ, Chaumeil PA, Hugenholtz P. 2022. GTDB: an ongoing census of bacterial and archaeal diversity through a phylogenetically

consistent, rank normalized and complete genome-based taxonomy. *Nucleic Acids Res.* [Internet] 50:D785–D794. Available from: https://dx.doi.org/10.1093/nar/gkab776

Quast C, Pruesse E, Yilmaz P, Gerken J, Schweer T, Yarza P, Peplies J, Glöckner FO. 2013. The SILVA ribosomal RNA gene database project: improved data processing and web-based tools. *Nucleic Acids Res.* [Internet] 41:D590–D596. Available from: https://academic.oup.com/nar/article/41/D1/D590/1069277

Ranwez V, Douzery EJP, Cambon C, Chantret N, Delsuc F. 2018. MACSE v2: Toolkit for the Alignment of Coding Sequences Accounting for Frameshifts and Stop Codons. *Mol. Biol. Evol.* [Internet] 35:2582–2584. Available from: https://dx.doi.org/10.1093/molbev/msy159

Rawlings TA, Collinst TM, Bieler R. 2003. Changing identities: tRNA duplication and remolding within animal mitochondrial genomes. *Proceedings of the National Academy of Sciences* [Internet] 100:15700–15705. Available from: /doi/pdf/10.1073/pnas.2535036100?download=true

Robinson DF, Foulds LR. 1981. Comparison of phylogenetic trees. *Math. Biosci.* 53:131–147.

Sayers EW, Cavanaugh M, Frisse L, Pruitt KD, Schneider VA, Underwood BA, Yankie L, Karsch-Mizrachi I. 2025. GenBank 2025 update. *Nucleic Acids Res.* [Internet] 53:D56–D61. Available from: https://dx.doi.org/10.1093/nar/gkae1114

Schoch CL, Ciufo S, Domrachev M, Hotton CL, Kannan S, Khovanskaya R, Leipe D, McVeigh R, O’Neill K, Robbertse B, et al. 2020. NCBI Taxonomy: a comprehensive update on curation, resources and tools. *Database* [Internet] 2020. Available from: https://academic.oup.com/database/article/doi/10.1093/database/baaa062/5881509

Strunecký O, Ivanova AP, Mareš J. 2023. An updated classification of cyanobacterial orders and families based on phylogenomic and polyphasic analysis. *J. Phycol.* [Internet] 59:12–51. Available from: https://onlinelibrary.wiley.com/doi/full/10.1111/jpy.13304

Tange O. 2025. GNU Parallel 20251222 ('Bondi') released [stable]. Available from: https://doi.org/10.5281/zenodo.18039569

Trgovec-Greif L, Hellinger HJ, Mainguy J, Pfundner A, Frishman D, Kiening M, Webster NS, Laffy PW, Feichtinger M, Rattei T. 2024. VOGDB—Database of Virus Orthologous Groups. *Viruses 2024, Vol. 16, Page 1191* [Internet] 16:1191. Available from: https://www.mdpi.com/1999-4915/16/8/1191/htm

Whelan S, Irisarri I, Burki F. 2018. PREQUAL: detecting non-homologous characters in sets of unaligned homologous sequences. *Bioinformatics* [Internet] 34:3929–3930. Available from: https://dx.doi.org/10.1093/bioinformatics/bty448

Yoo AB, Jette MA, Grondona M. 2003. SLURM: Simple Linux Utility for Resource Management. *Lecture Notes in Computer Science (including subseries Lecture Notes in Artificial Intelligence and Lecture Notes in Bioinformatics)* [Internet] 2862:44–60. Available from: https://link.springer.com/chapter/10.1007/10968987_3

Yu D, Ren Y, Uesaka M, Beavan AJS, Muffato M, Shen J, Li Y, Sato I, Wan W, Clark JW, et al. 2024. Hagfish genome elucidates vertebrate whole-genome duplication events and their evolutionary consequences. *Nat. Ecol. Evol.* [Internet] 8:519–535. Available from: https://doi.org/10.1038/s41559-023-02299-z

Supplementary Tables

Table S1. AliFilter model performance. Comparisons between manually filtered test datasets and the AliFilter moder trained on dataset 9 (full dataset). A: accuracy; MCC: Matthews correlation coefficient; C: model confidence; AUC: area under the receiver operating characteristic curve.

	**Overall**				**Alignment-wise (1st quartile – median – 3rd quartile)**			
**Test dataset**	A	MCC	C	AUC	A	MCC	C	AUC
**1 (n=139)**	0.98	0.96	0.95	1.00	0.99 - 0.99 - 1.00	0.95 - 0.98 - 1.00	0.94 - 0.96 - 0.97	1.00 - 1.00 - 1.00
**2 (n=10)**	0.99	0.98	0.97	1.00	0.99 - 0.99 - 1.00	0.96 - 0.97 - 1.00	0.96 - 0.96 - 0.97	1.00 - 1.00 - 1.00
**3 (n=10)**	0.97	0.91	0.95	1.00	0.96 - 0.98 - 0.99	0.80 - 0.88 - 0.91	0.93 - 0.96 - 0.96	0.99 - 1.00 - 1.00
**4 (n=13)**	0.97	0.93	0.84	1.00	0.96 - 0.98 - 0.99	0.91 - 0.95 - 0.97	0.83 - 0.86 - 0.91	1.00 - 1.00 - 1.00
**5 (n=10)**	0.96	0.92	0.90	0.99	0.95 - 0.97 - 0.99	0.90 - 0.95 - 0.98	0.88 - 0.92 - 0.94	0.99 - 1.00 - 1.00
**6 (n=10)**	0.99	0.95	0.95	1.00	0.97 - 0.99 - 1.00	0.92 - 0.96 - 1.00	0.95 - 0.97 - 0.98	1.00 - 1.00 - 1.00
**7 (n=10)**	0.98	0.95	0.95	1.00	0.99 - 1.00 - 1.00	0.94 - 0.99 - 1.00	0.96 - 0.97 - 0.99	1.00 - 1.00 - 1.00
**8 (n=14)**	0.98	0.96	0.94	1.00	0.97 - 0.98 - 0.99	0.88 - 0.93 - 0.97	0.91 - 0.93 - 0.96	0.99 - 1.00 - 1.00
**9 (n=216)**	0.98	0.96	0.94	1.00	0.98 - 0.99 - 1.00	0.93 - 0.97 - 0.99	0.94 - 0.96 - 0.97	1.00 - 1.00 - 1.00
**12 (n=35)**	0.98	0.96	0.90	1.00	0.97 - 0.98 - 0.99	0.94 - 0.97 - 0.98	0.87 - 0.88 - 0.90	1.00 - 1.00 - 1.00
**13 (n=73)**	0.98	0.93	0.85	1.00	0.97 - 0.99 - 0.99	0.90 - 0.96 - 0.97	0.84 - 0.87 - 0.90	1.00 - 1.00 - 1.00

Table S2. Computational effort required for a phylogenomic analysis. Alignments from dataset 1 were filtered using various tools and used for a maximum-likelihood phylogenomic analysis in IQ-TREE v2.3.6 (Minh et al. 2020). Each analysis was repeated three times. The tree length is the sum of the lengths of all branches in the tree, expressed as substitutions per site.

Filtering	Alignment length	Distinct patterns	RAM required	Runtime	Tree length	Median runtime	Median tree length
**None**	82564	61296	263 GB	51.96 h<br>50.16 h<br>54.36 h	87.37<br>87.15<br>87.11	51.96 h	87.15
**AliFilter**	51684	45786	197 GB	32.81 h<br>33.68 h<br>40.15 h	57.70<br>57.70<br>57.72	33.68 h	57.70
**BMGE**	49295	43407	186 GB	36.70 h<br>29.82 h<br>31.24 h	47.71<br>47.71<br>47.71	31.24 h	47.71
**ClipKIT**	65308	57190	246 GB	41.60 h<br>50.65 h<br>48.24 h	85.10<br>85.04<br>85.13	48.24 h	85.10
**Gblocks**	43247	37393	161 GB	31.26 h<br>31.09 h<br>33.92 h	35.91<br>35.90<br>35.90	31.26 h	35.90
**Noisy**	51602	46235	199 GB	36.87 h<br>31.70 h<br>34.22 h	87.85<br>87.90<br>87.84	34.22 h	87.85
**trimAl**	48668	42755	184 GB	27.84 h<br>31.31 h<br>33.01 h	46.82<br>46.79<br>46.83	31.31 h	46.82
**Manual**	53295	47386	203 GB	34.42 h<br>31.84 h<br>41.59 h	62.55<br>62.53<br>62.57	34.42 h	62.55

Table S3. Comparisons between AliFilter models. Alignments manually filtered by different authors were used to train AliFilter models, and each model was tested against a test dataset filtered by the same person or by a different person. A: accuracy; MCC: Matthews correlation coefficient; C: model confidence; AUC: area under the receiver operating characteristic curve. In the 'Comparison' column, 'test set' refers to the manually filtered test alignments, while 'model' refers to the same test alignments, filtered by an AliFilter model trained using the training and validation alignments manually filtered by the specified author. *: These rows show the similarity (A) and correlation (MCC) between two manually filtered datasets, for which the model confidence score (C) and the AUC are not computed.

	Dataset	Comparison		A	MCC	C	AUC
*	10	GB (test set)	ERRM (test set)	0.95	0.90	-	-
	10	GB (model)	GB (test set)	0.98	0.97	0.93	1.00
	10	GB (model)	ERRM (test set)	0.95	0.90	0.93	0.99
	10	ERRM (model)	ERRM (test set)	0.95	0.89	0.82	0.99
	10	ERRM (model)	GB (test set)	0.98	0.95	0.82	1.00
*	11	GB (test set)	FC (test set)	0.85	0.73	-	-
	11	GB (model)	GB (test set)	0.98	0.95	0.94	1.00
	11	GB (model)	FC (test set)	0.85	0.74	0.94	0.98
	11	FC (model)	FC (test set)	0.98	0.95	0.96	0.98
	11	FC (model)	GB (test set)	0.83	0.71	0.96	0.99

Table S4. Test results for models trained using a dataset and evaluated using a different dataset. For each combination of training/validation dataset and test dataset, the accuracy (A), Matthews correlation coefficient (MCC), and the area under the receiver operating characteristic curve (AUC) are shown as A / MCC / AUC. Values lower than 0.85 are shown in **bold**. Comparisons where all datasets have been filtered by the same author are underlined.

Training and validation datasets (author)	Test dataset (author): 1 (GB)	8 (GB)	10 (GB)	11 (GB)	10 (ERRM)	11 (FC)
**8 (GB)**	0.98 / 0.95 / 0.98	0.97 / 0.94 / 0.98	0.96 / 0.91 / 0.95	0.97 / 0.95 / 0.98	0.94 / 0.87 / 0.93	**0.83** / **0.70** / 0.86
**9 (GB)**	0.98 / 0.96 / 1.00	0.98 / 0.96 / 1.00	0.98 / 0.95 / 1.00	0.98 / 0.95 / 1.00	0.95 / 0.89 / 0.99	**0.84** / **0.72** / 0.95
**10 (GB)**	0.98 / 0.96 / 1.00	0.97 / 0.95 / 1.00	0.98 / 0.97 / 1.00	0.98 / 0.95 / 0.99	0.95 / 0.90 / 0.99	**0.83** / **0.70** / 0.90
**11 (GB)**	0.97 / 0.93 / 1.00	0.98 / 0.96 / 1.00	0.94 / 0.87 / 1.00	0.98 / 0.95 / 1.00	0.92 / **0.82** / 0.98	0.85 / **0.74** / 0.98
**10 (ERRM)**	0.98 / 0.96 / 1.00	0.97 / 0.94 / 1.00	0.98 / 0.95 / 1.00	0.98 / 0.95 / 0.99	0.95 / 0.89 / 0.99	**0.83** / **0.70** / 0.91
**11 (FC)**	0.94 / 0.89 / 0.97	0.88 / **0.77** / 0.98	0.93 / **0.84** / 0.92	**0.83** / **0.71** / 0.98	0.91 / **0.79** / 0.90	0.98 / 0.95 / 0.99

Table S5. AliFilter models trained using other tools. Each tool was used to filter all alignments in dataset 9. Training, validation and testing were then performed on these alignments. A: accuracy; MCC: Matthews correlation coefficient; C: model confidence; AUC: area under the receiver operating characteristic curve.

Tool	$\boldsymbol{A}$	$\boldsymbol{MCC}$	$\boldsymbol{C}$	$\boldsymbol{AUC}$
**BMGE**	0.98	0.97	0.99	0.99
**ClipKIT**	0.89	0.72	0.75	0.95
**Gblocks**	0.95	0.90	0.98	0.96
**Noisy**	0.76	0.46	0.95	0.76
**trimAl**	0.84	0.70	0.99	0.86

Supplementary Figures

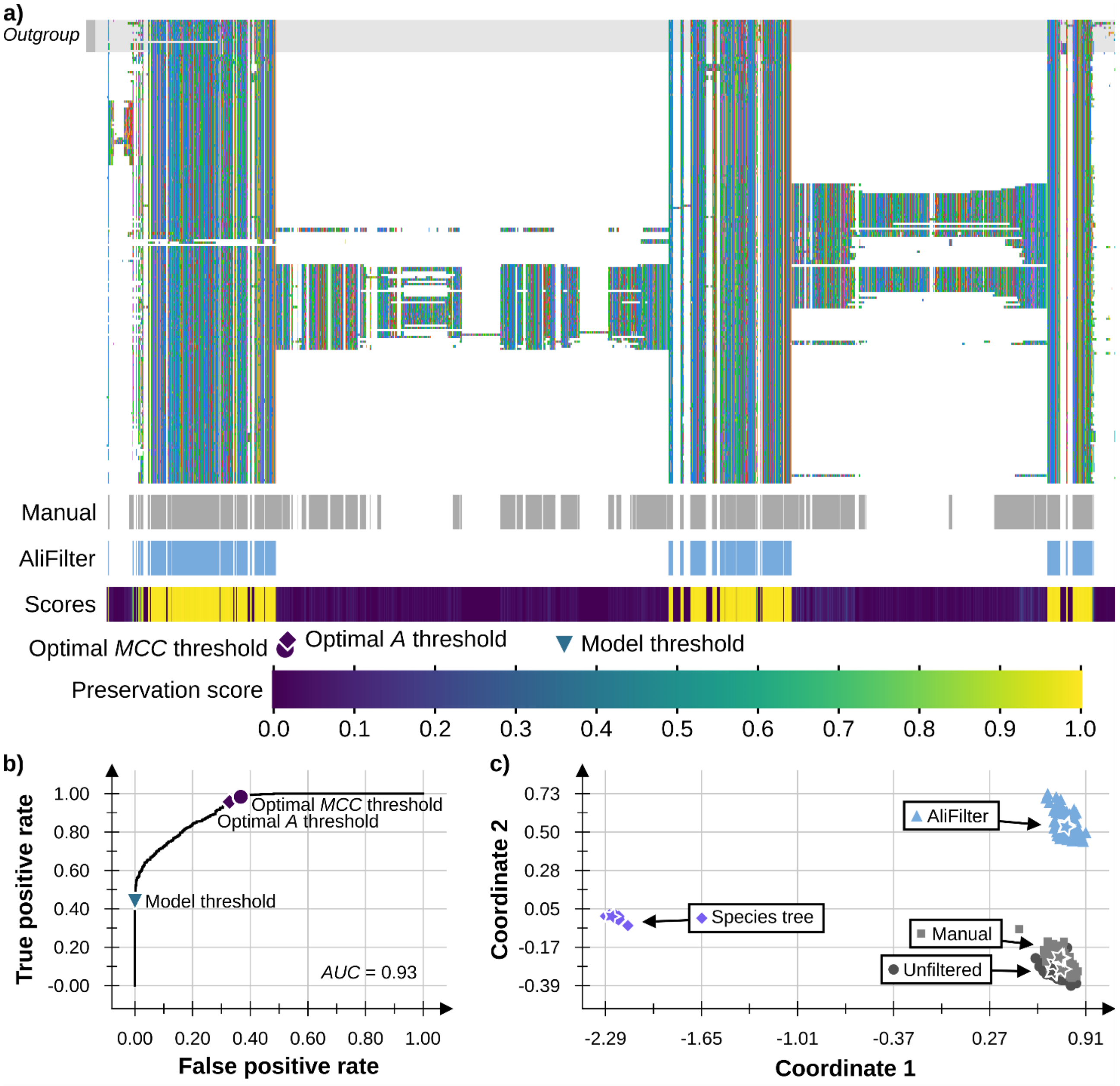

Figure S1. a) Protein sequence alignment for *dnaB* orthologs. This is one of two sequence alignments where AliFilter and the manual filtering approach produce significantly different results. Sequence regions that are conserved only across some sequences are incorrectly deleted by AliFilter. **b) Receiver Operating Characteristic (ROC) curve.** The curve represents the true positive rate (TPR) and false positive rate (FPR) for different filtering thresholds. The TPR and FPR corresponding to the threshold

value used by the model ($A = 0.68$, $MCC = 0.50$) are highlighted, as well as the values corresponding to the thresholds that maximise the accuracy ($A = 0.84$, $MCC = 0.67$) or the Matthews Correlation Coefficient ($A = 0.83$, $MCC = 0.68$) for this specific alignment. AUC: Area Under the (ROC) Curve. **c) Visualisation of the tree space according to the Frobenius distance.** Maximum-likelihood trees across three replicates for each alignment are highlighted by stars, while bootstrap replicates are shown by coloured symbols. For this gene, the alignment filtered by AliFilter produces different trees than the unfiltered alignment and the manually filtered alignment (which are similar to each other). However, the species tree computed using the full dataset ('Unfiltered' in main text **Fig. 5b**) differs significantly from the gene trees.

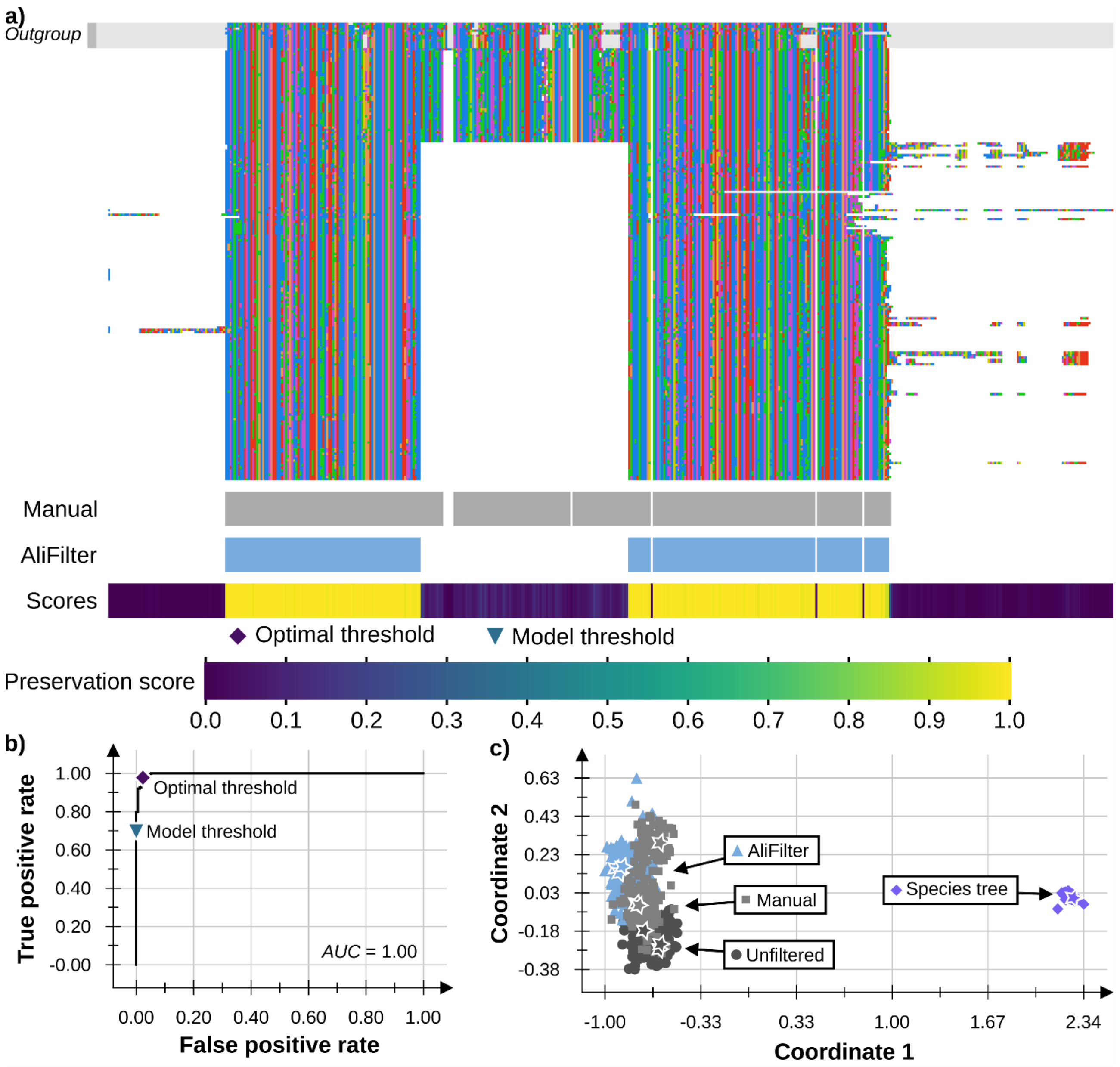

Figure S2. a) Protein sequence alignment for *tsf* orthologs. This is one of two sequence alignments where AliFilter and the manual filtering approach produce significantly different results. Sequence regions that are conserved only across some sequences are incorrectly deleted by AliFilter. **b) Receiver Operating Characteristic (ROC) curve.** The curve represents the true positive rate (TPR) and false positive rate (FPR) for different filtering thresholds. The TPR and FPR corresponding to the threshold value used by the model ($A = 0.81$, $MCC = 0.68$) are highlighted, as well as the values corresponding to the threshold that maximises both the accuracy and the Matthews

Correlation Coefficient for this specific alignment ($A = 0.99$, $MCC = 0.97$). AUC: Area Under the (ROC) Curve. **c) Visualisation of the tree space according to the Frobenius distance.** Maximum-likelihood trees across three replicates for each alignment are highlighted by stars, while bootstrap replicates are shown by coloured symbols. For this gene, even though the alignment filtered by AliFilter is significantly different than the manually filtered alignment, both of them produce similar trees to the unfiltered alignment. However, the species tree computed using the full dataset (‘Unfiltered’ in main text **Fig. 5b**) differs significantly from the gene trees.

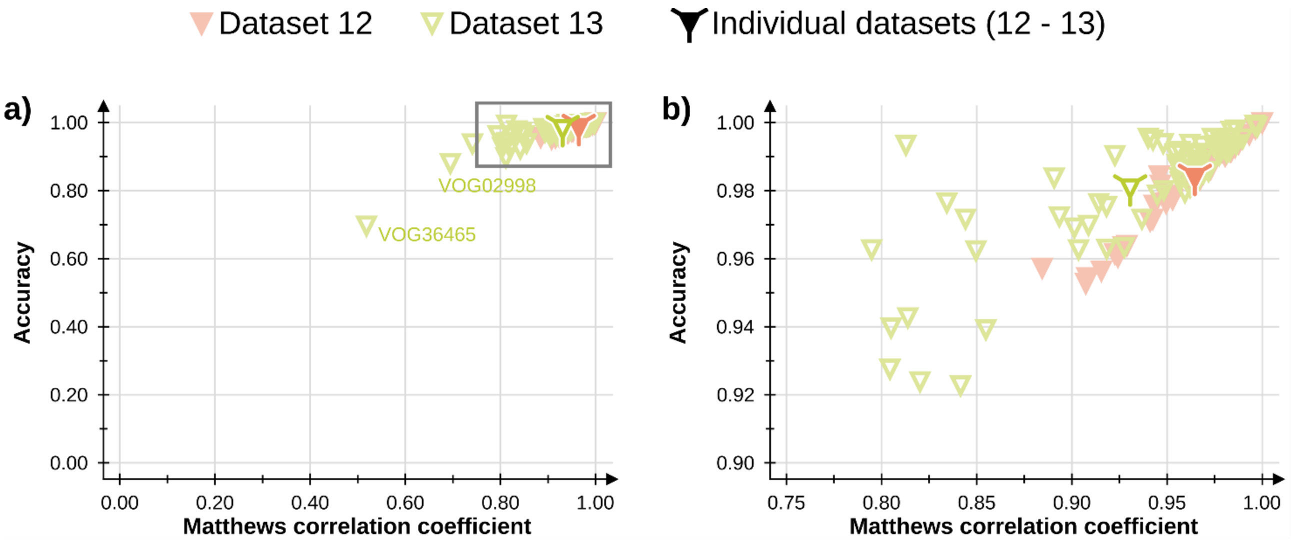

Figure S3. Performance metrics for the main AliFilter model on mitochondrial and viral sequences. a) Each plotted point represents one of 108 alignments (35 for the mitochondrial Dataset 12, and 73 for the viral Dataset 13), while three-pointed stars represent the overall results for the two datasets. Alignments with Matthews correlation coefficient < 0.70 are highlighted; like *dnaB* and *tsf* (**Fig. S1, S2**), this is due to long insertions that are preserved by the manual approach and deleted by AliFilter. The grey square identifies the area shown in more detail in part b. **b)** Zoomed-in view of the datapoints with Accuracy ≥ 0.90 and Matthews correlation coefficient ≥ 0.75. Dataset numbers as in **Table 2**.

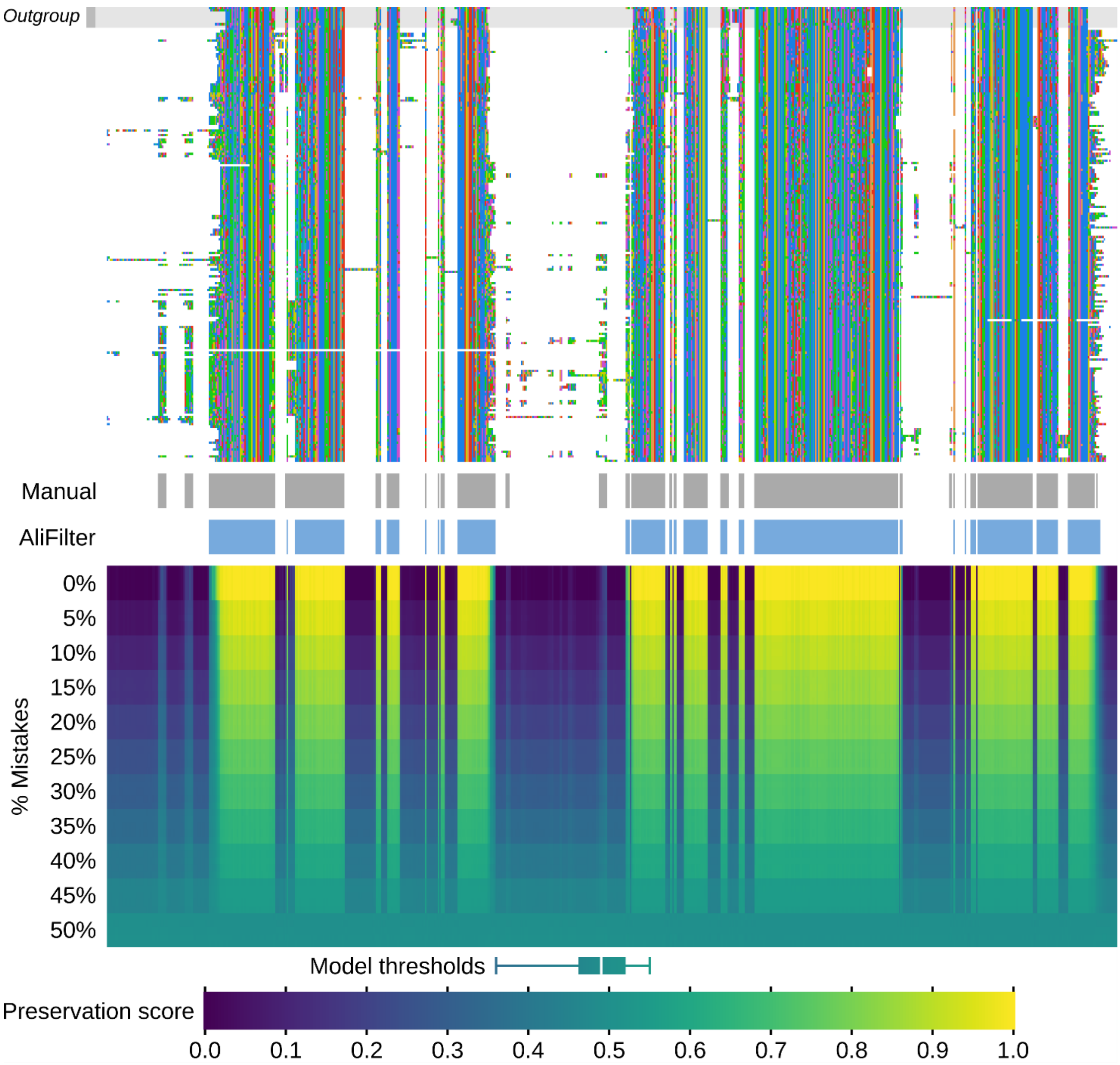

Figure S4. Effect of mistakes in the training and validation datasets on the preservation scores. The protein sequence alignment shown here is the same as main text **Figure 1**. The preservation score for each alignment column obtained using models trained and validated with varying proportions of mistakes is shown at the bottom of the figure. As the proportion of mistakes increases, scores draw closer to 50%. However, the model is still able to distinguish between columns that should be preserved and those that should be discarded until the proportion of mistakes reaches 50% (at which point the training/validation datasets essentially consist of random assignments).

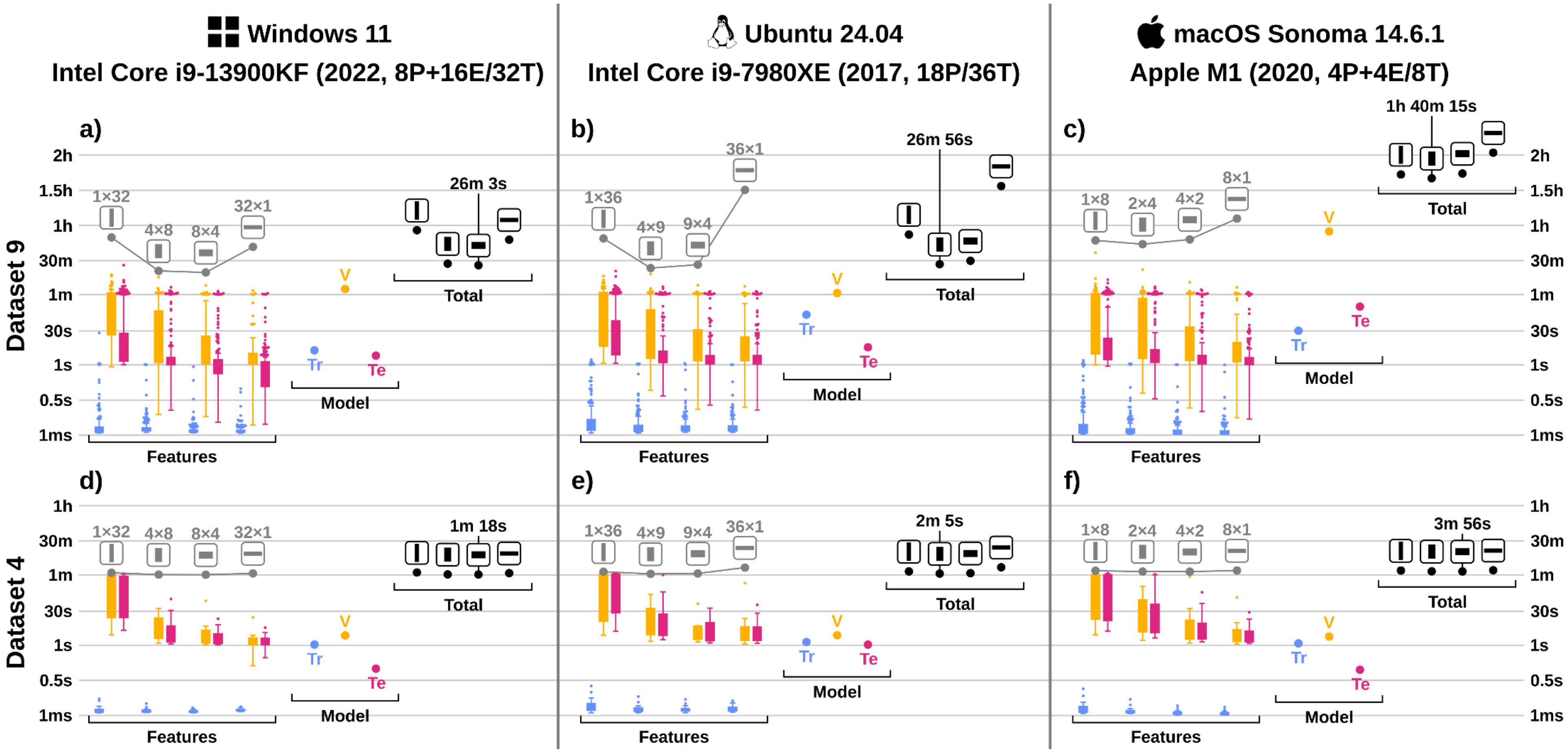

Figure S5. Model training benchmarks. These plots show the amount of time necessary to train an AliFilter model on three different machines, divided between feature computation, model training (Tr), validation (V) and testing (Te). The full Dataset 9 was used for **a**, **b** and **c**, whereas only Dataset 4 was used for **d**, **e**, and **f**. In **a** and **d**, the analysis was run on a desktop computer built in 2022, running Windows 11 and equipped with an Intel Core i9-13900KF processor with 8 performance (P) cores, 16 efficiency (E) cores, and 32 logical processors (T). For **b** and **e**, we used a machine from 2017, running Ubuntu Server 24.04 on an Intel Core i9-7980XE with 18 physical

and 36 logical processors. In **c** and **f**, we used an Apple MacBook Pro (13-inch, 2020) with an Apple M1 processor with 4 P cores, 4 E cores, and 8 logical processors. Feature computation from annotated alignments is an embarrassingly parallel process (Herlihy et al. 2021), and for each analysis we show runtime estimates from four distinct parallelisation strategies, listed as $T \times J$, where T is the number of threads per job, and J is the maximum number of concurrent jobs (e.g., 4×8 indicates that up to eight jobs were allowed to run in parallel, utilising four threads each). In all cases, the values of T and J were chosen so that the product $T \cdot J$ would be equal to the number of logical processors available. On Windows, job scheduling was performed using the Parallel feature of the ForEach-Object cmdlet in PowerShell v7.5.4 (Higinbotham 2019), while the Slurm Workload Manager (Yoo et al. 2003) was used on Linux and the GNU parallel utility (Tange 2025) was used on macOS. The box plots represent the feature computation execution time for each alignment, with outliers as swarm plots (validation and test alignments require a longer time, because bootstrap replicates are used in these cases); the grey points represent the overall execution time of the feature computation step for each parallelisation strategy (including any scheduling overhead). The total amount of time necessary to fully train and test the model is shown in black, highlighting the value for the optimal parallelisation strategy in each case. Note that the vertical scale is not linear.

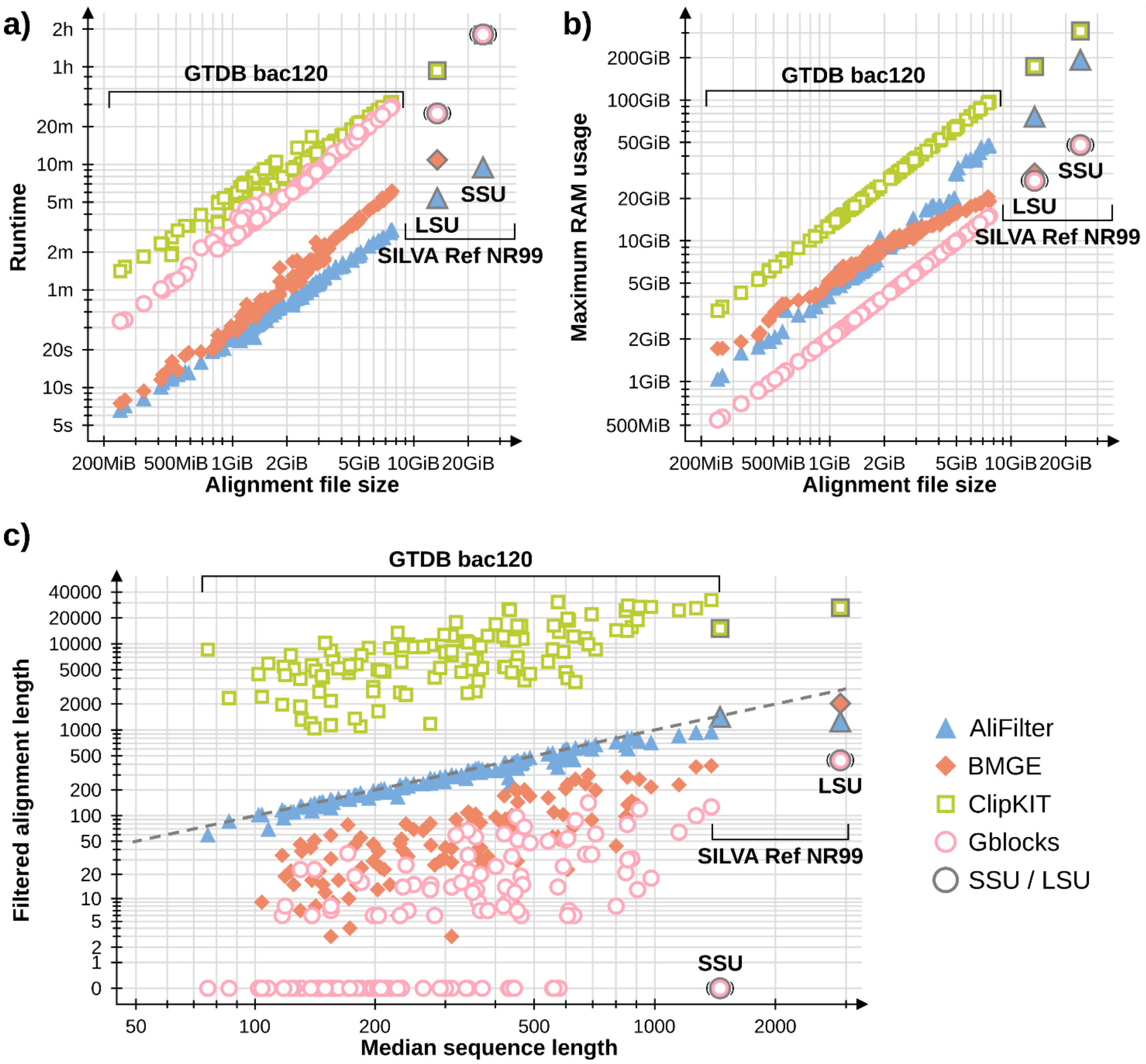

Figure S6. Alignment filtering benchmarks on large alignments. Each point represents a single alignment, with the two SILVA Ref NR99 alignments (SSU and LSU) highlighted by a grey border. **a) Execution time of the filtering step for each alignment using different tools, plotted as a function of the alignment file size**. AliFilter is consistently faster than BMGE, ClipKIT and Gblocks, and even the largest alignment is filtered in less than 10 minutes. BMGE is slightly slower, while Gblocks and ClipKIT are much slower. **b) Maximum RAM usage as a function of the alignment file size.** AliFilter requires a similar amount of RAM as BMGE for alignments files up to 5GiB, but this increases for larger alignments. Gblocks requires less RAM than the other tools, while ClipKIT requires

more. **c) Length of the filtered alignment as a function of the median length of unaligned sequences**. The dashed line represents points where $y = x$. AliFilter generally produces alignments whose length is similar to the length of unaligned sequences, while ClipKIT produces considerably larger alignments. BMGE and especially Gblocks alignments are much shorter, and in 48 out of 122 cases (39%), Gblocks produced completely empty alignments. Noisy and trimAl are excluded from this analysis, as they did not produce any output after running for six hours. BMGE crashed with a Java heap space out of memory error when analysing the SILVA Ref NR99 SSU alignment. For Gblocks, data points for the SILVA Ref NR99 SSU and LSU alignments are shown in parentheses, because the program reported an incorrect number of columns in the original alignment (interestingly, the program did not report any error message and produced a seemingly normal output). Runtime (“Elapsed (wall clock) time”) and maximum RAM usage (“Maximum resident set size”) were determined using the GNU time utility. Benchmarks were conducted under Rocky Linux 8.9 on compute nodes equipped with an Intel Xeon Gold 6226R CPU (64 logical processors) and 320GB of RAM. When filtering, a single node was exclusively allocated to each alignment, and the relevant command-line options were used to instruct AliFilter and ClipKIT to use all 64 logical processors (to take advantage of this, version 2.7.0 of ClipKIT was used; BMGE, Gblocks, Noisy, and trimAl lack this option).

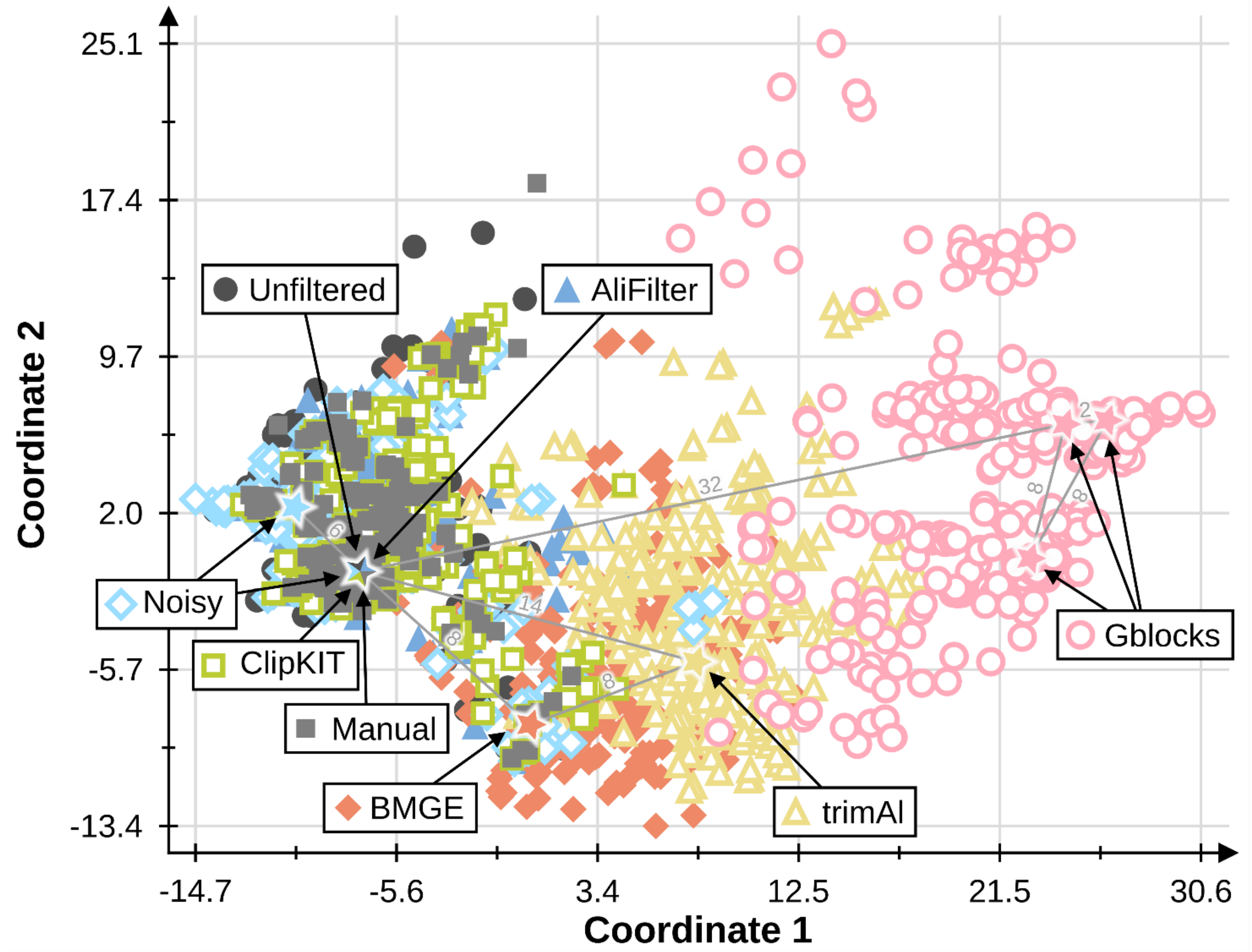

Figure S7. Visualisation of the tree space according to the Robinson-Foulds (RF) distance. A classical metric dimensional scaling approach was used to visualise the RF distances between maximum-likelihood and ultrafast bootstrap replicates built using IQ-TREE v2.3.6 (Minh et al. 2020) on alignments filtered with different tools. Each symbol on the plot represents a single topology; stars represent maximum-likelihood (ML) estimates (three for each tool, but always overlapping except for Gblocks and Noisy). ML topologies for the unfiltered alignment, manually filtered alignment, AliFilter, ClipKIT, and Noisy (two out of three replicates) are identical. The light grey lines and numbers show the RF distance between select pairs of topologies.

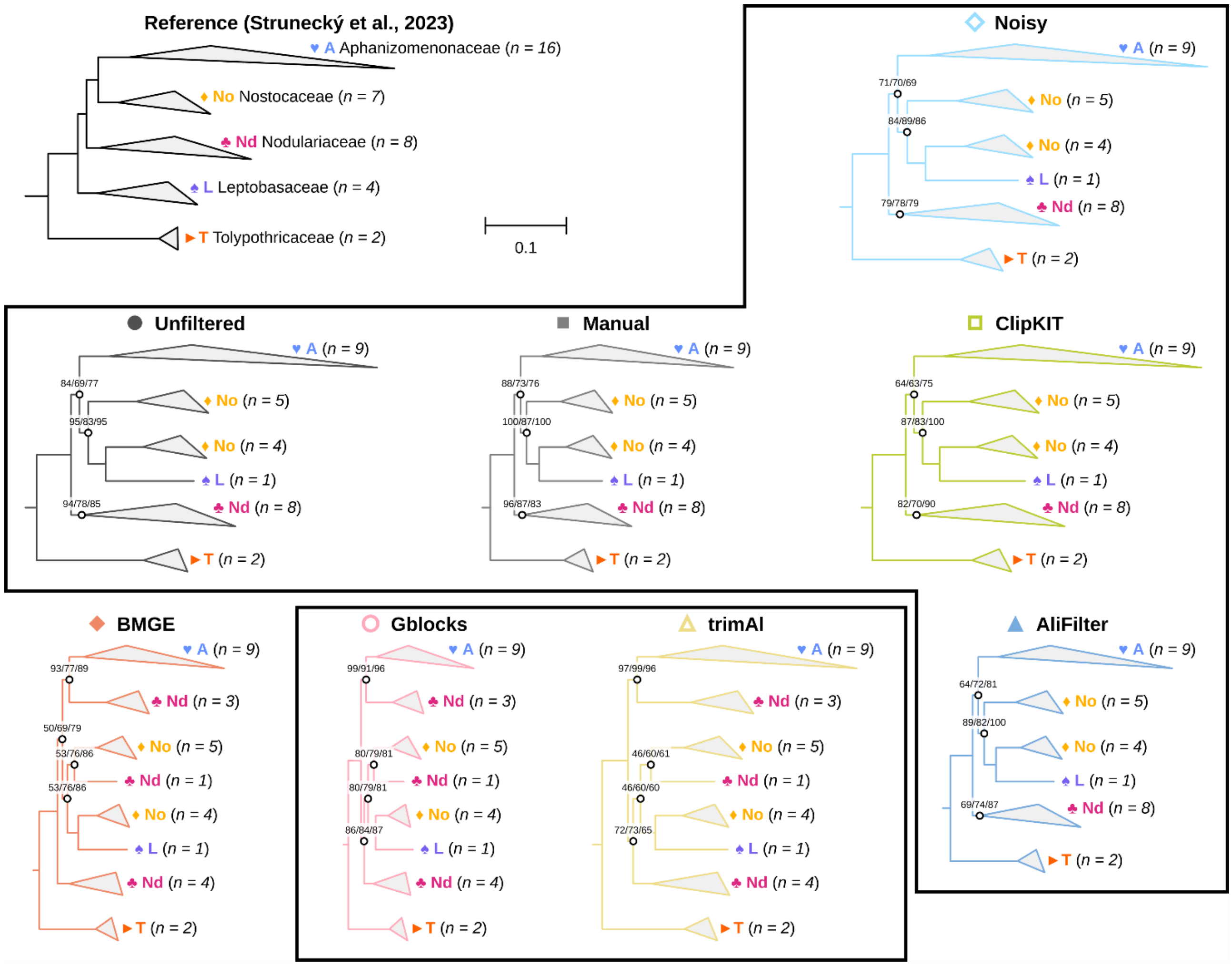

Figure S8. Comparison between phylogenetic trees of Cyanobacteria obtained using alignments filtered with different tools. Tree topologies for a group in the Nostocales order of Cyanobacteria (where most of the differences shown in **Figure S7** are found) are compared between a reference tree (Strunecký et al. 2023) and the results obtained using Dataset 1 filtered with each tool, as well as the unfiltered alignments and the manually filtered ones. Branch lengths in units of substitutions per site are to scale between the trees and represent the median of three replicates; ultrafast bootstrap support values lower than 100% across the three replicates are highlighted. Trees with the same topology are surrounded by black lines. In all cases, when using Dataset 1 the family Leptobasaceae is nested within the Nostocaceae, likely because only a single strain from this family is included in the dataset. With varying support, the unfiltered and manually filtered alignments, as well as AliFilter, Noisy and ClipKIT, correctly recover monophyly of the

Nodulariaceae; in contrast, using alignments filtered with BMGE, Gblocks, or trimAl results in polyphyletic Nodulariaceae.

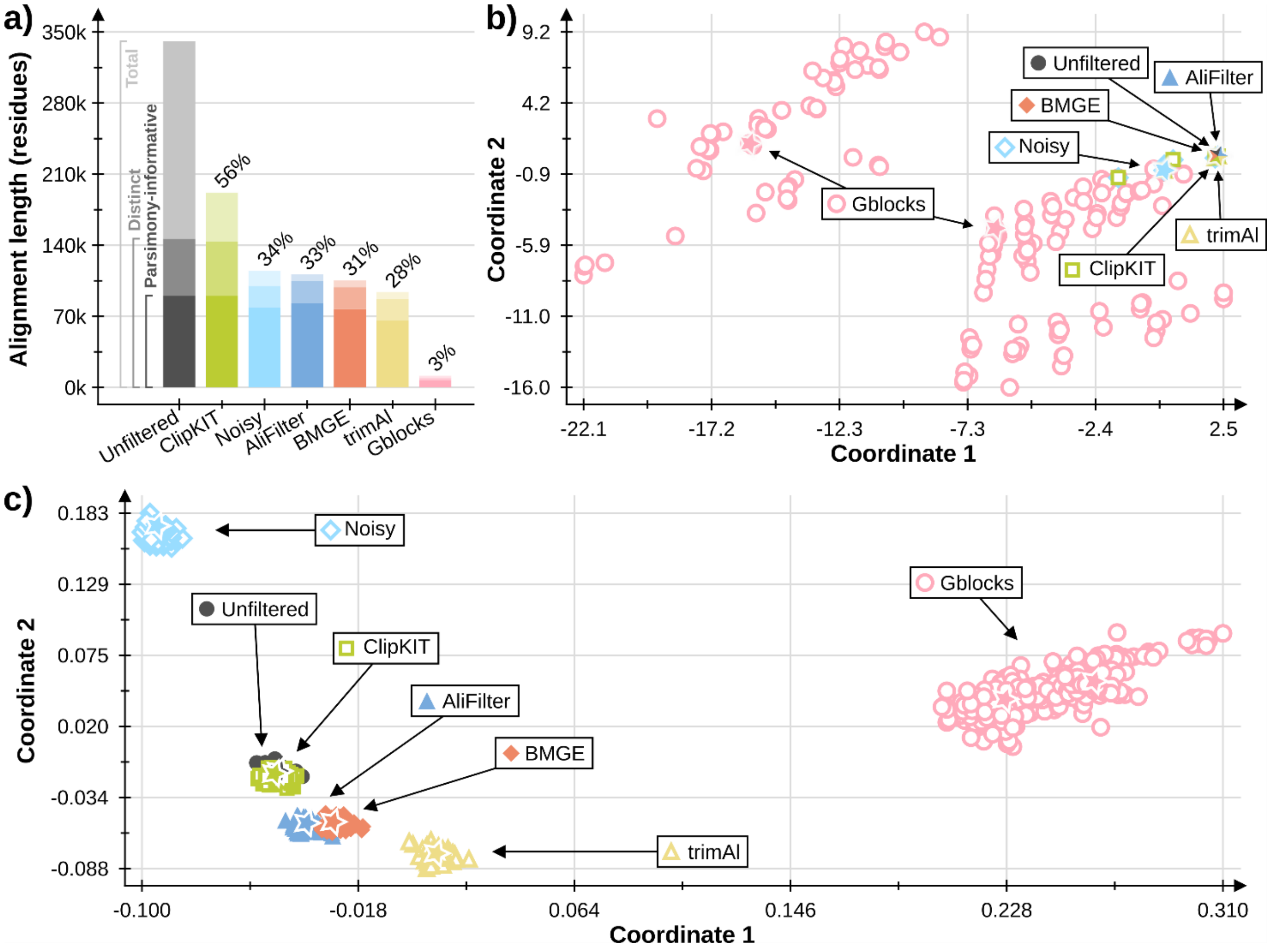

Figure S9. Effect of alignment filtering on a phylogenomics-level analysis of animals. a) Preserved proportions of alignment columns. Out of the total 340 811 columns in the unfiltered alignment, ClipKIT preserved 56%, Gblocks preserved 3%, while the other tools preserved around 30%. The number of parsimony-informative sites and distinct patterns (as reported by IQ-TREE) are also highlighted. **b) Visualisation of the tree space according to the Robinson-Foulds distance**. Each symbol on the plot represents a single ultrafast bootstrap replicate tree (300 for each tool); except for Gblocks, most symbols are concentrated behind star symbols, which represent the maximum-likelihood trees (three for each tool, often overlapping). **c) Visualisation of the tree space according to the Frobenius distance**. Symbols represent individual trees as in part **b**.

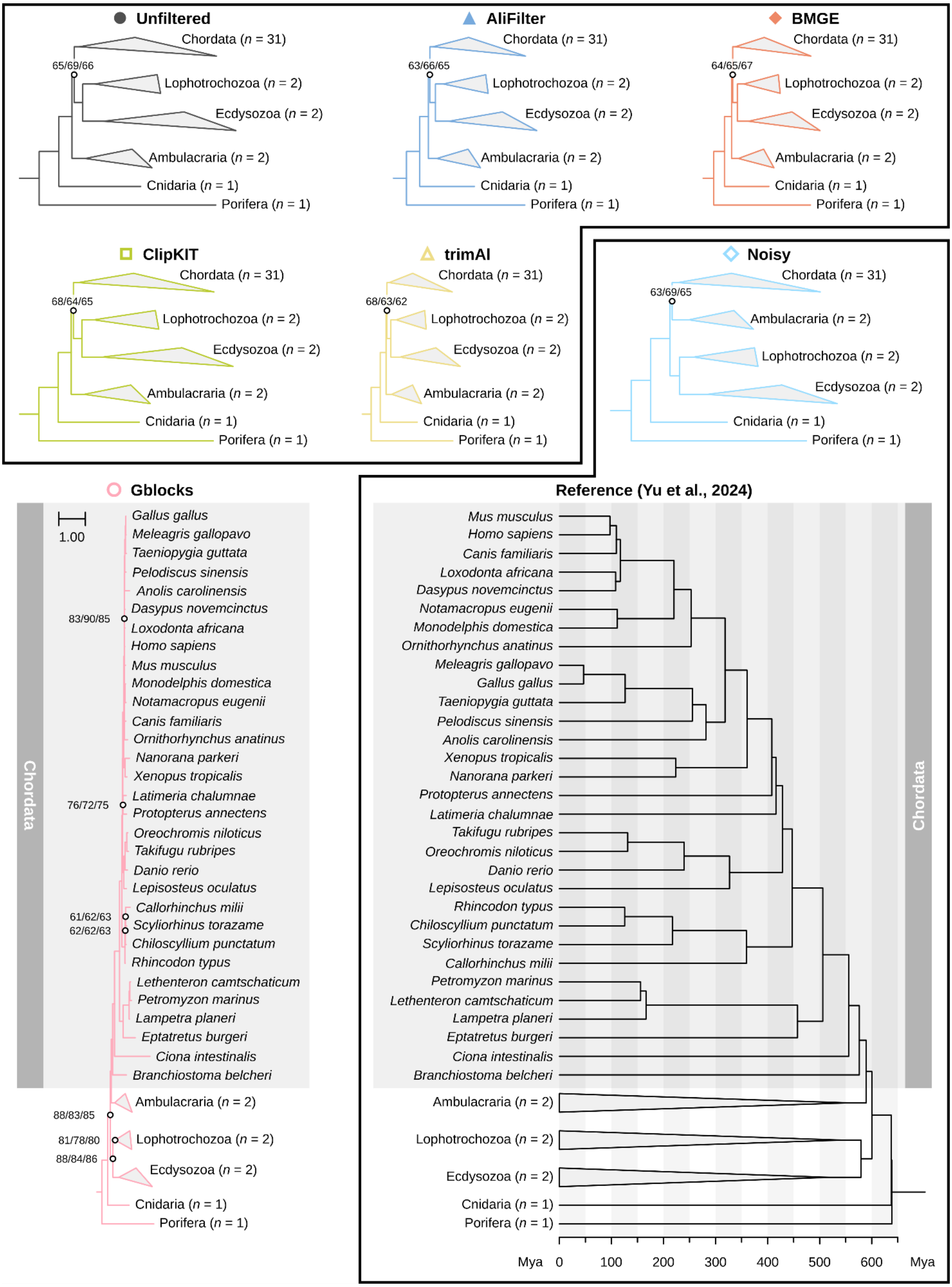

Figure S10. Comparison between phylogenetic trees of animals obtained using alignments filtered with different tools. The tree topologies obtained using unfiltered

alignments and alignments filtered by AliFilter, BMGE, ClipKIT and trimAl are identical, and differ from the one produced by Noisy only at a single node: Noisy recovers monophyletic Deuterostomia (i.e., Chordata + Ambulacraria), while the other tools recover Centroneuralia (Chordata + Protostomia (Kapli et al. 2025)), in all cases with low support. The tree topology recovered by Noisy is identical to the reference tree (Yu et al. 2024); for all tools except Gblocks, relationships within the phylum Chordata are also identical. In contrast to other tools, the Gblocks tree topology is highly uncertain, but shows 100% bootstrap support for monophyletic Deuterostomia. In all cases, both Chordata and Ambulacraria are recovered as monophyletic with 100% support. Except for the reference (which is a time-calibrated molecular clock tree), branch lengths in units of substitutions per site are to scale between the trees and represent the median of three replicates; ultrafast bootstrap support values lower than 100% are highlighted.

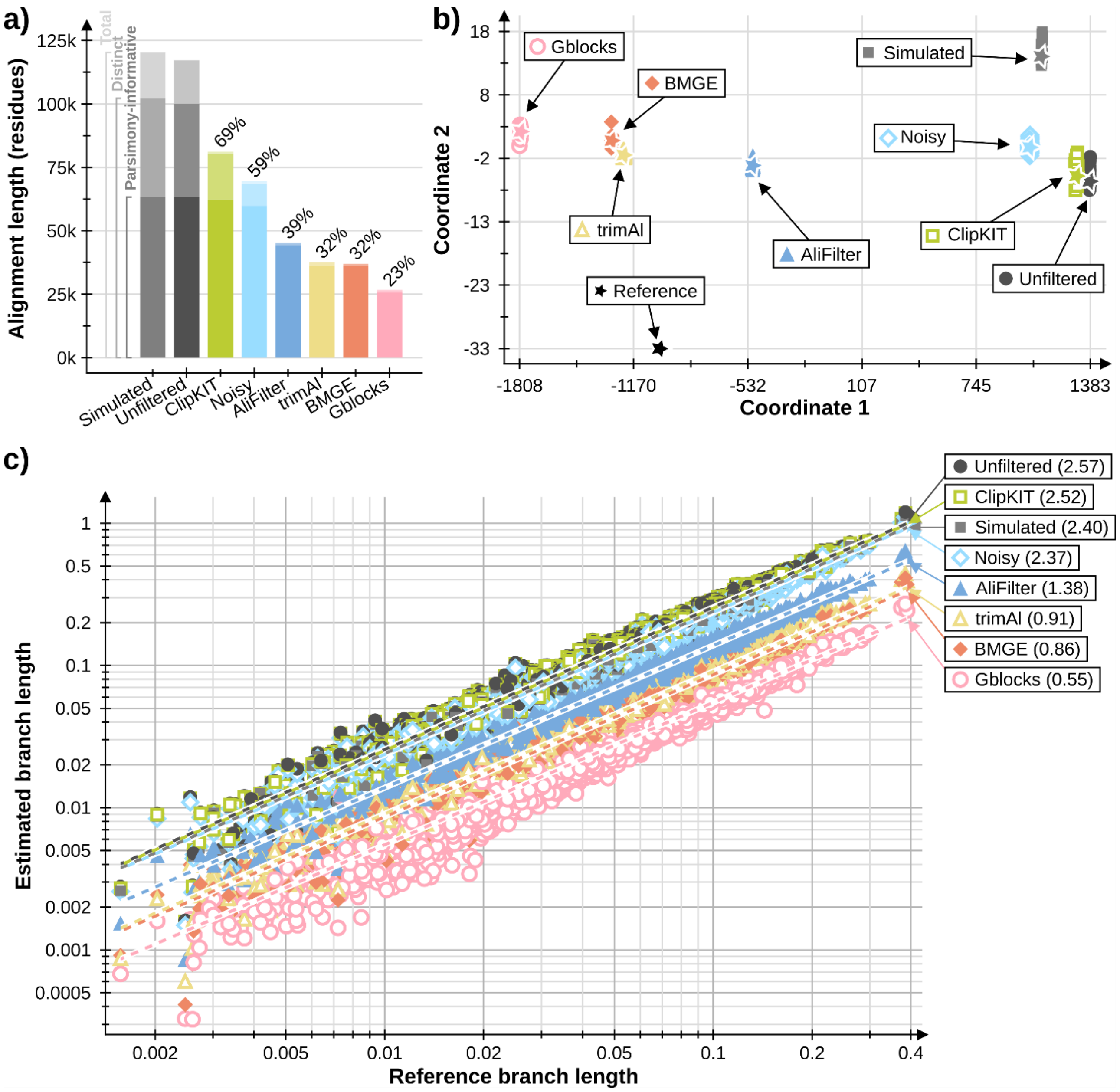

Figure S11. Effect of alignment filtering on a simulated dataset. a) Preserved proportions of alignment columns. The simulated alignment contained 120 214 columns, while the re-aligned sequences totalled 117 218 columns. The percentages represent the proportion of re-aligned columns preserved by each tool. The number of parsimony-informative sites and distinct patterns (as reported by IQ-TREE) are also highlighted. **b) Visualisation of the tree space according to the Frobenius distance**. Each symbol on the plot represents a single ultrafast bootstrap replicate tree (300 for each tool); most symbols are concentrated behind star symbols, which represent the maximum-likelihood trees (three for each tool, often overlapping). The maximum-likelihood tree

topologies for all tools were identical to the reference tree. **c) Comparison between branch lengths in the reference tree and in maximum-likelihood trees**. Each symbol represents a single branch; the horizontal axis shows the branch length in the reference tree, while the vertical axis represents the branch length in the maximum-likelihood tree inferred using alignments filtered with each tool (median of three replicates). Each tool preserves a different proportion of slow- and fast-evolving sites, resulting in different branch length estimates; however, these are generally proportional to the branch lengths in the reference tree. The dashed lines represent linear regressions through the origin, which appear as parallel lines due to the logarithmic scale of the plot. Slope values are reported in parentheses next to the name of each tool and $R^2 \approx 0.98$ in all cases.

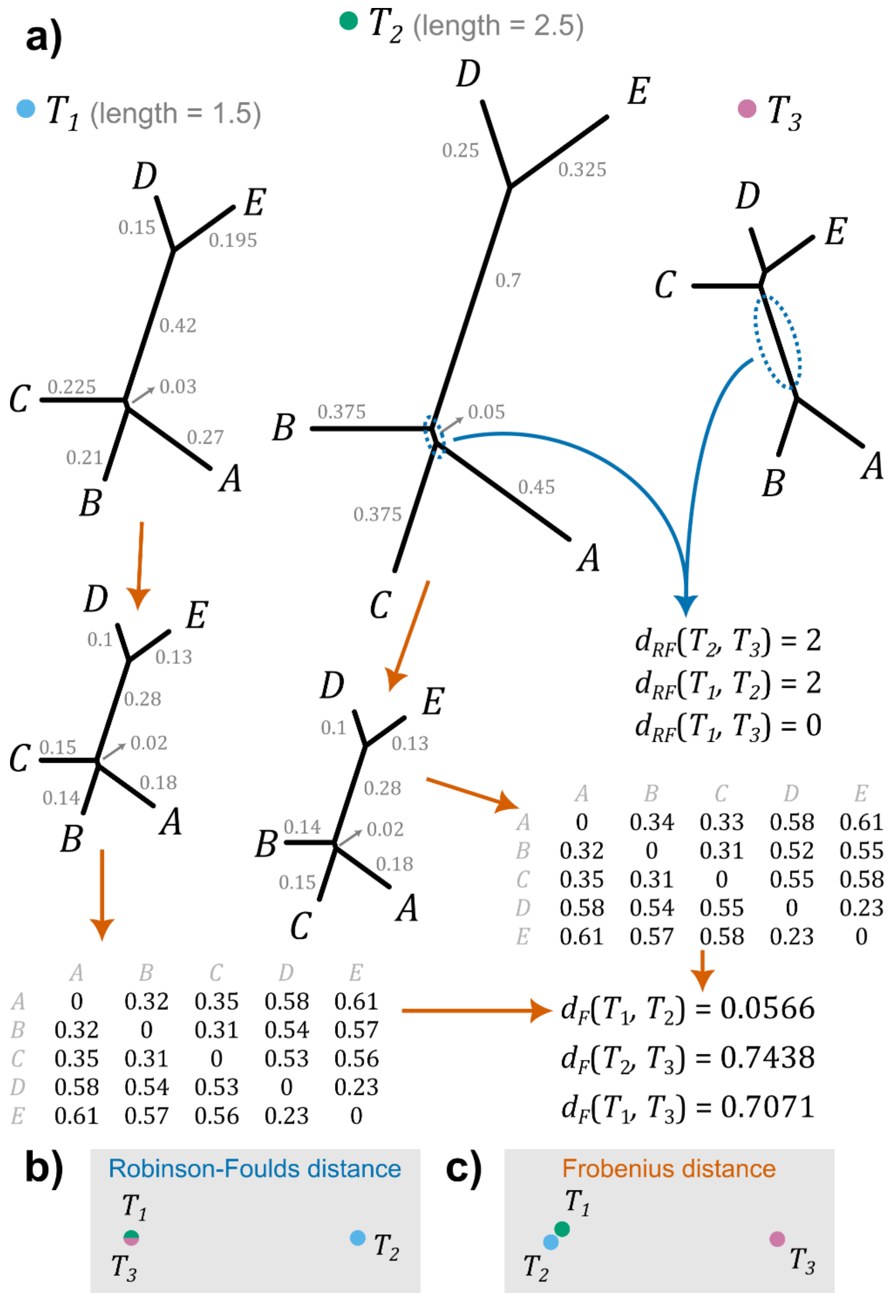

Figure S12. Phylogenetic tree distances. a) Three trees are being compared. To compute the Robinson-Foulds distance (d_{RF}) between T_2 and T_3, splits that are present in one tree but not the other are identified (dashed ellipses); $d_{RF}(T_2, T_3)$ is the total number of such splits. To compute the Frobenius distance (d_F), each tree is first normalised, then converted into a patristic distance matrix (orange arrows). The distance between two trees is the Frobenius distance between the corresponding matrices. **b)** Visualisation of the tree space according to the Robinson-Foulds distance. T_1 and T_3 are represented by the same point. **c)** Visualisation of the tree space according to the Frobenius distance.
