# Supplementary material for: AliFilter: a machine learning approach to alignment filtering
Source: Mol Biol Evol. 2026 Apr 10;43(4):msag097. doi: 10.1093/molbev/msag097 (PMC13108598; doi:10.1093/molbev/msag097)
Supplement: msag097_Supplementary_Data [file msag097_supplementary_data.zip › alignment.report.pdf]

Alignment report

Created by AliFilter version 1.0.0 on 24th Nov, 2024 at 18:55.

MD5 checksum of the model file used to filter this alignment: `E95D52E3F38227FDA87BC60EBBE59E34`

This report may be included in any analysis using this alignment.

This report is machine-readable. If the report file is called `report.pdf`, you can export the model to a file called `model.json` by running:

- On a Unix machine:

  ```
  grep -a "@model" report.pdf | sed "s/@model//g" > model.json
  ```

- On a Windows machine (within a PowerShell environment):

  ```
  findstr "@model" report.pdf | %{$_ -replace "@model",""} > model.json
  ```

Input data analysis

The input alignment contained 715 columns of amino acids. For each alignment column, 6 features were computed (**Table 1**), which were analysed in a Principal Component Analysis (PCA) and in a Linear Discriminant Analysis (LDA).

Table 1. Alignment features. The table lists the features that have been computed for each alignment column, including a brief description and the observed range, mean and standard deviation (SD) for each of them.

Name	Description	Values
Gap proportion	Proportion of sequences that have a gap in the column.	*Range*: 0 - 0.9949<br>*Mean*: 0.4759<br>*SD*: 0.4716
Percent identity	Frequency of the most common residue in the alignment column, excluding gaps.	*Range*: 0.0051 - 1<br>*Mean*: 0.3512<br>*SD*: 0.3841
Distance from extremity	Number of residues between the column and the closest extremity (start or end) of the alignment.	*Range*: 0 - 357<br>*Mean*: 178.2503<br>*SD*: 103.2016
Entropy	Shannon entropy for the residue frequencies in the column, excluding gaps.	*Range*: 0 - 2.7745<br>*Mean*: 0.8712<br>*SD*: 0.8275
Gap proportion (±1)	Average of the proportion of gaps between the column, 1 preceding column(s), and 1 subsequent columns.	*Range*: 0 - 0.9949<br>*Mean*: 0.4759<br>*SD*: 0.4472
Gap proportion (±2)	Average of the proportion of gaps between the column, 2 preceding column(s), and 2 subsequent columns.	*Range*: 0 - 0.9949<br>*Mean*: 0.4759<br>*SD*: 0.4327

A PCA (**Fig. 1**) uses a linear transformation to transform the data to a coordinate system where each coordinate (component) explains as much of the variance of the data as possible, while being orthogonal to the previous components. This is useful to show the distribution of the input data, but a PCA, on its own, cannot be used to decide whether an alignment column should be preserved or not.

An LDA (**Fig. 2**) also uses a linear transformation to project the data to a different coordinate space, but in this case each component attempts to explain as much of the difference between the two classes of data (“preserved” or “deleted”) as possible. While the transform used to create the PCA plot (**Fig. 1**) was computed using only the input data, the LDA transform used to create **Figure 2** was computed during the model training step.

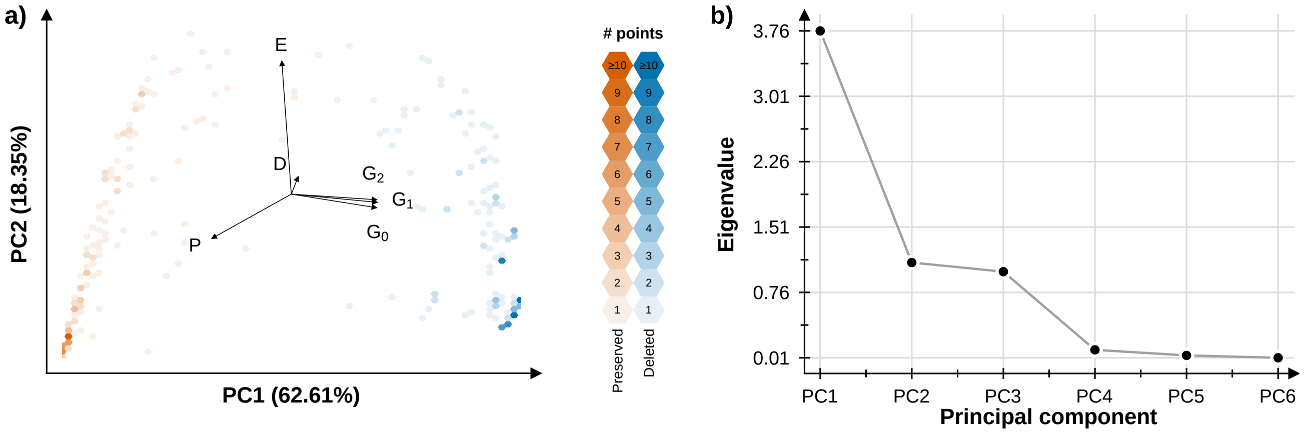

Figure 1. Results of the PCA. a) Biplot showing the density of input data columns in function of the principal component values and the component loadings. Columns that were preserved in the filtered alignment are shown in ⬣ orange, while deleted columns are shown in ⬣ blue. Component abbreviations are *PC1*: Principal component 1; *PC2*: Principal component 2; G_0: Gap proportion; *P*: Percent identity; *D*: Distance from extremity; *E* : Entropy; G_1: Gap proportion (±1); G_2: Gap proportion (±2). **b)** Scree plot showing the eigenvalue (amount of explained variance) corresponding to each principal component.

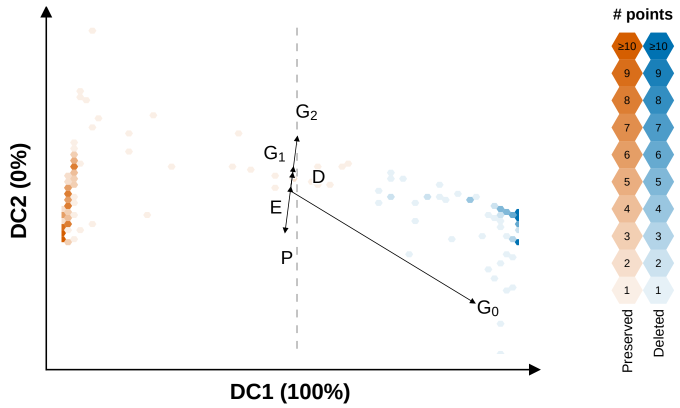

Figure 2. Results of the LDA. The biplot shows the density of input data columns in function of the discriminant component values and the projections of the original features in the LDA space. Columns that were preserved in the filtered alignment are shown in ⬣ orange, while deleted columns are shown in ⬣ blue. The - - - dashed line represents the intersection of the discriminant hyperplane with the 2D plane shown in the plot. Component abbreviations are *DC1*: Discriminant component 1; *DC2*: Discriminant component 2; the rest as in **Figure 1a**.

Results

The data described above were analysed with a logistic model, which uses a linear combination of the feature values for each column to determine the log-odds that the column be preserved. The log-odds were converted to a preservation score (ranging from 0 to 1), and columns with a preservation score lower than a specified threshold (for this model, 0.36) were deleted.

Using this approach, 372 columns (52.03%) were preserved, while 343 columns (47.97%) were deleted (**Fig. 3**). The distribution of the preservation scores for each column computed using this model is shown in **Figure 4**.

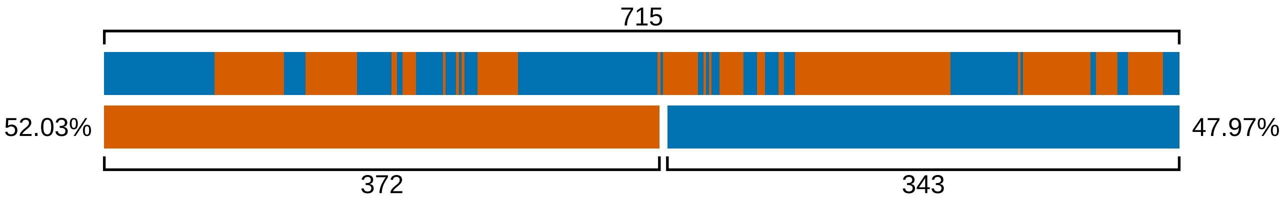

Figure 3. Proportion of preserved columns. The upper part of the figure shows the alignment columns that were preserved (in ■ orange) or deleted (in ■ blue) in the filtered alignment. The bottom part of the figure shows the proportion of preserved and deleted columns in the filtered alignment.

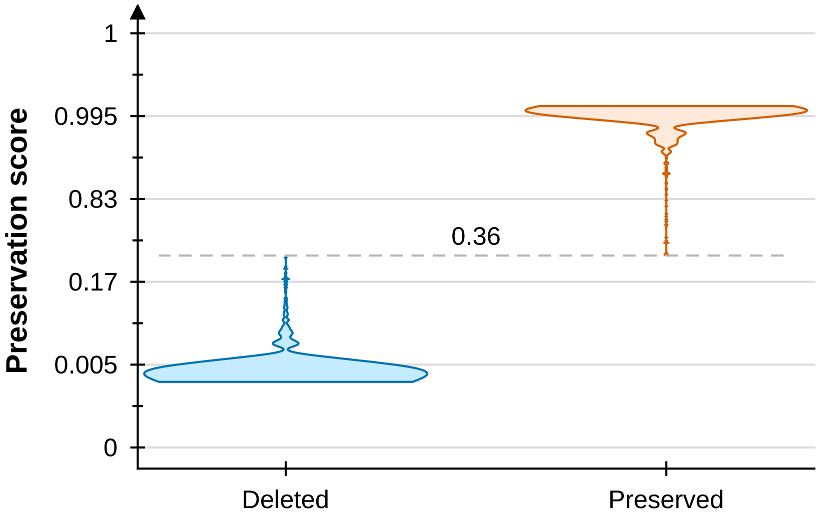

Figure 4. Logistic model results. This violin plot shows the distribution of the preservation score according to the logistic model for columns that were deleted (in ● blue, on the left) or preserved (in ● orange, on the right) in the filtered alignment. The - - - dashed line represents the 0.36 threshold that determines whether a column is preserved or deleted according to the model.

The threshold value (here, 0.36) determines which columns are deleted or preserved. Alignment columns with a score lower than the threshold are deleted, while those with a score higher than or equal to the threshold are preserved. **Figure 5** shows the proportion of columns that are deleted as a function of the threshold value. If the model is able confidently classify the columns in the alignment, this line should be almost flat for a wide range of values between 0 and 1. This can be summarised using the model confidence score C, which is defined as:

$$C = 1 - \frac{4}{n} \sum_{i=1}^{n} s_i \cdot (1 - s_i)$$

Where n is the number of columns in the alignment and s_i is the confidence score for column i (ranging between 0 and 1). For a model performing confident assignments, this score should be close to 1. In this case, it is 0.9155.

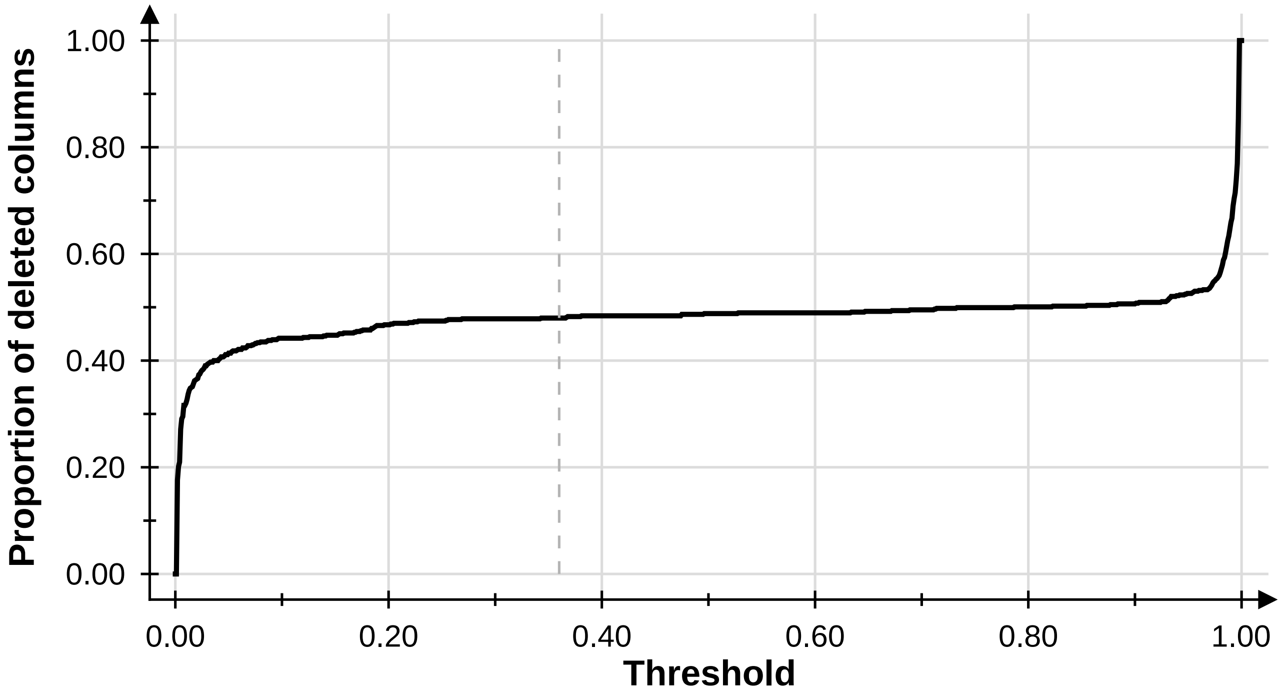

Figure 5. Proportion of deleted columns as a function of the threshold value. The line chart shows the proportion of columns in the input alignment that are deleted when a certain threshold value is used. The - - - dashed line represents the 0.36 preservation threshold.
